# Fern genomes elucidate land plant evolution and cyanobacterial symbioses

**DOI:** 10.1038/s41477-018-0188-8

**Published:** 2018-07-02

**Authors:** Fay-Wei Li, Paul Brouwer, Lorenzo Carretero-Paulet, Shifeng Cheng, Jan de Vries, Pierre-Marc Delaux, Ariana Eily, Nils Koppers, Li-Yaung Kuo, Zheng Li, Mathew Simenc, Ian Small, Eric Wafula, Stephany Angarita, Michael S. Barker, Andrea Bräutigam, Claude dePamphilis, Sven Gould, Prashant S. Hosmani, Yao-Moan Huang, Bruno Huettel, Yoichiro Kato, Xin Liu, Steven Maere, Rose McDowell, Lukas A. Mueller, Klaas G. J. Nierop, Stefan A. Rensing, Tanner Robison, Carl J. Rothfels, Erin M. Sigel, Yue Song, Prakash R. Timilsena, Yves Van de Peer, Hongli Wang, Per K. I. Wilhelmsson, Paul G. Wolf, Xun Xu, Joshua P. Der, Henriette Schluepmann, Gane K.-S. Wong, Kathleen M. Pryer

**Affiliations:** 1 Boyce Thompson Institute, Ithaca, NY USA; 2 Plant Biology Section, Cornell University, Ithaca, NY USA; 3 Molecular Plant Physiology Department, Utrecht University, Utrecht, the Netherlands; 4 Bioinformatics Institute Ghent and Department of Plant Biotechnology and Bioinformatics, Ghent University, Ghent, Belgium; 5 VIB Center for Plant Systems Biology, Ghent, Belgium; 6 BGI-Shenzhen, Beishan Industrial Zone, Shenzhen, China; 7 Department of Biochemistry and Molecular Biology, Dalhousie University, Halifax, Nova Scotia Canada; 8 Laboratoire de Recherche en Sciences Végétales, Université de Toulouse, CNRS, UPS, Castanet Tolosan, France; 9 Department of Biology, Duke University, Durham, NC USA; 10 Department of Plant Biochemistry, Cluster of Excellence on Plant Sciences, Heinrich Heine University Düsseldorf, Dusseldorf, Germany; 11 Department of Ecology and Evolutionary Biology, University of Arizona, Tucson, AZ USA; 12 Department of Biological Science, California State University, Fullerton, CA USA; 13 ARC Centre of Excellence in Plant Energy Biology, School of Molecular Sciences, The University of Western Australia, Crawley, Western Australia Australia; 14 Department of Biology, Huck Institutes of the Life Sciences, Pennsylvania State University, University Park, PA USA; 15 Faculty of Biology, Bielefeld University, Bielefeld, Germany; 16 Institute for Molecular Evolution, Heinrich Heine University Düsseldorf, Dusseldorf, Germany; 17 Taiwan Forestry Research Institute, Taipei, Taiwan; 18 Max Planck Genome Centre Cologne, Max Planck Institute for Plant Breeding, Cologne, Germany; 19 Institute for Sustainable Agro-ecosystem Services, University of Tokyo, Tokyo, Japan; 20 Geolab, Faculty of Geosciences, Utrecht University, Utrecht, the Netherlands; 21 Faculty of Biology, University of Marburg, Marburg, Germany; 22 Department of Biology, Utah State University, Logan, UT USA; 23 University Herbarium and Department of Integrative Biology, University of California, Berkeley, CA USA; 24 Department of Biology, University of Louisiana, Lafayette, LA USA; 25 Department of Biochemistry, Genetics and Microbiology, University of Pretoria, Pretoria, South Africa; 26 Department of Biological Sciences, Department of Medicine, University of Alberta, Edmonton, Alberta Canada

**Keywords:** Plant symbiosis, Natural variation in plants, Phylogenetics, Comparative genomics, Genome evolution

## Abstract

Ferns are the closest sister group to all seed plants, yet little is known about their genomes other than that they are generally colossal. Here, we report on the genomes of *Azolla filiculoides* and *Salvinia cucullata* (Salviniales) and present evidence for episodic whole-genome duplication in ferns—one at the base of ‘core leptosporangiates’ and one specific to *Azolla*. One fern-specific gene that we identified, recently shown to confer high insect resistance, seems to have been derived from bacteria through horizontal gene transfer. *Azolla* coexists in a unique symbiosis with N_2_-fixing cyanobacteria, and we demonstrate a clear pattern of cospeciation between the two partners. Furthermore, the *Azolla* genome lacks genes that are common to arbuscular mycorrhizal and root nodule symbioses, and we identify several putative transporter genes specific to *Azolla*–cyanobacterial symbiosis. These genomic resources will help in exploring the biotechnological potential of *Azolla* and address fundamental questions in the evolution of plant life.

## Abstract (web summary)

The genomes of two fern species, *Azolla filiculoides* and *Salvinia cucullata*, are reported and provide insights into fern-specific whole-genome duplications, fern-specific insect-resistant gene evolution and fern–cyanobacterial symbiosis.

## Main

The advent of land plants ~474–515 Myr ago^1^ led to complex vegetational innovations that shaped emerging terrestrial and freshwater ecosystems. Bryophytes, lycophytes, ferns and gymnosperms dominated the global landscape before the ecological radiation of flowering plants 90 Myr ago^2^. The first complete plant genome sequence (*Arabidopsis thaliana*) was published in 2000^3^, followed by reference genomes for all other major lineages of green plants, except ferns. A dearth of genomic information from this entire lineage has limited not only our knowledge of fern biology but also the processes that govern the evolution of land plants.

The relatively small genome (0.75 Gb)^4^ of *Azolla* is exceptional among ferns, a group that is notorious for genomes as large as 148 Gb^5^ and averaging 12 Gb^6^. *Azolla* is one of the fastest-growing plants on the planet, with demonstrated potential to be a significant carbon sink. Data from the Arctic Ocean show that, ~50 Myr ago, in middle-Eocene sediments, an abundance of fossilized *Azolla* characterizes an ~800,000-year interval known as the ‘*Azolla* event’^7^. This period coincides with the shift from the early Eocene greenhouse world towards our present icehouse climate, suggesting that *Azolla* had a role in abrupt global cooling by sequestering atmospheric carbon dioxide^8^. *Azolla* is also remarkable in harbouring an obligate, N_2_-fixing cyanobacterium, *Nostoc azollae*, within specialized leaf cavities. Because of this capability, *Azolla* has been used as ‘green manure’ for over 1,000 years to bolster rice productivity in Southeast Asia^9^. The *Azolla* symbiosis is unique among plant–bacterial endosymbiotic associations because the cyanobiont is associated with the fern throughout its life cycle, being vertically transmitted during sexual reproduction to subsequent generations^10^. In all other land plant symbiotic associations, the relationship must be renewed each generation. The *Nostoc* symbiont is not capable of autonomous growth and its genome shows clear signs of reduction, with several housekeeping genes lost or pseudogenized^11^. With a fossil record that extends back to the mid-Cretaceous period, *Azolla* probably shares a ~100-Myr-old co-evolutionary relationship with *Nostoc*^12^.

**Fig. 1:**
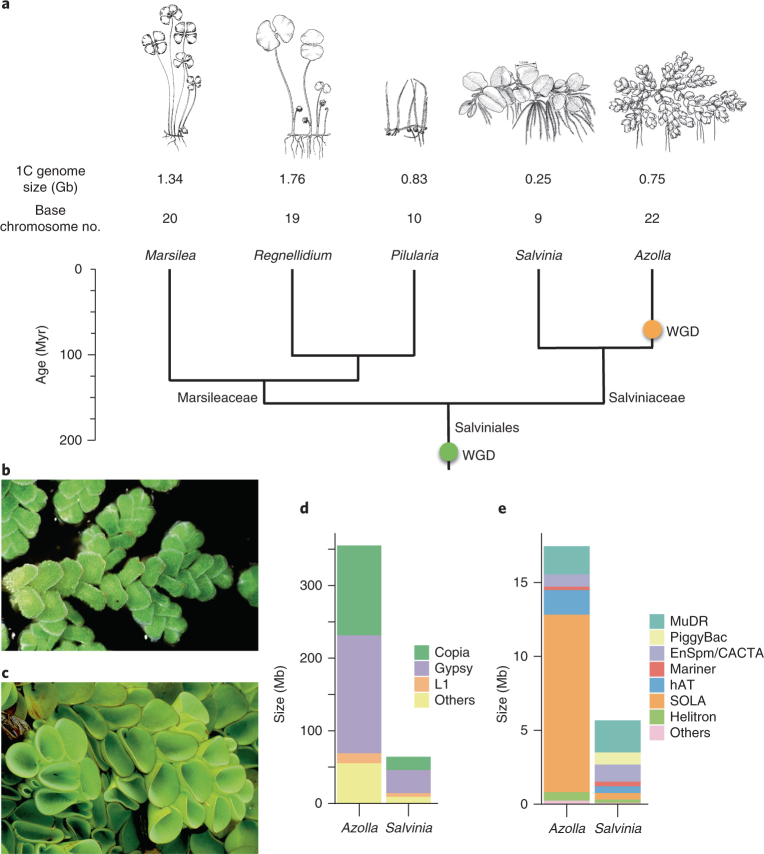
Genome size evolution in Salviniales. **a**, Members of Salviniales have smaller genome sizes than other ferns (averaging 1C = 12 Gb)^6^. Two whole-genome duplication (WGD) events identified in this study were mapped onto the phylogeny, with divergence time estimates obtained from Testo and Sundue^129^. **b**,**c**, Whole genomes were assembled from *A.* *filiculoides* (**b**) and *S.* *cucullata* (**c**). **d**,**e**, The genome of *S.* *cucullata* has substantially reduced levels of RNA (**d**) and DNA (**e**) transposons compared to *A.* *filiculoides*. Image in panel **c** courtesy of P.-F. Lu.

To better understand genome size evolution in *Azolla* and its closely related lineages, we obtained genome size estimates for all five genera of Salviniales (Supplementary Table 1). We found them to be at least an order of magnitude smaller than any other fern species (Fig. 1a), and, most notably, the genome of *Salvinia cucullata*, which belongs to the sister genus to *Azolla*, is only 0.26 Gb, the smallest genome size ever reported in ferns. This unanticipated discovery afforded us the opportunity to include a second fern genome for comparison.

## Results

### Genome assembly and annotation

To gain insight into fern genome evolution, as well as plant–cyanobacterial symbioses, we sequenced the genomes of *A.* *filiculoides* (Fig. 1b) and *S.* *cucullata* (Fig. 1c) using Illumina and PacBio technologies. The assembled *Azolla* and *Salvinia* genomes have N50 contig size of 964.7 Kb and 719.8 Kb, respectively. The BUSCO (Benchmarking Universal Single-Copy Orthologs) assessment and Illumina read-mapping results indicate high completeness for both assemblies (Supplementary Fig. 1 and Supplementary Table 2). We identified 20,201 and 19,914 high-confidence gene models in *Azolla* and *Salvinia*, respectively, that are supported by transcript evidence or had significant similarity to other known plant proteins (Supplementary Figs. 1–3, Supplementary Table 3 and Supplementary Discussion). *Salvinia* genes are much more compact, with a mean intron length half of that in *Azolla* (Supplementary Fig. 1). In addition to introns, differences in the repetitive content explain some of the nearly threefold difference in genome size. *Azolla* has more of every major category of repeat, but 191 Mb of the 233-Mb difference in the total repetitive content are made of retroelements, especially Gypsy and Copia long terminal repeat retrotransposons (LTR-RTs; Fig.1d and Supplementary Fig. 4). DNA transposon profiles are similar for the two ferns except that *Azolla* has substantially more SOLA elements than does *Salvinia* (Fig. 1e).

### Insights into gene family evolution in land plants

The genomes of *Azolla* and *Salvinia* offer a new opportunity to examine the evolution of plant genes and gene families across all Viridiplantae (land plants plus green algae). We classified genes into orthogroups from 23 genomes (12 angiosperms, 2 gymnosperms, 2 ferns, 1 lycophyte, 2 mosses, 2 liverworts, 1 charophyte and 1 chlorophyte; Supplementary Table 5) and reconstructed the gene family evolution—gain, loss, expansion and contraction—across the green tree of life (Supplementary Fig. 5 and Supplementary Table 5). To investigate the origin of genes linked to seed development, we examined orthogroups containing 48 transcription factors that express exclusively in *Arabidopsis* seeds^13^. Homologues of 39 of them were detected in ferns or other seed-free plants, indicating that many seed transcription factors were present before the origin of seeds (Supplementary Table 6). Similarly, only a handful of transcription factor families arose along the branch that led to seed plants (Supplementary Table 7); rather than relying on entirely novel transcription factors, it seems instead that an expansion of pre-existing transcription factor families had a greater role in seed plant evolution^14^. Indeed, ancestral gene number reconstructions of MADS-intervening keratin-like and C-terminal (MIKC)-type MADS box genes (orthogroup 23) show that these important developmental regulators more than doubled in number from 15 in the ancestral vascular plant to 31 in the ancestral euphyllophyte (here, Salviniales plus seed plants; Supplementary Table 5).

In a recent study on the evolution of plant transcription-associated proteins, which include transcription factors and transcriptional regulators^14^, ferns were exclusively represented by the *Pteridium aquilinum* transcriptome. The finding that the transcriptional regulator Polycomb group EZ (PcG_EZ) was lost in ferns is corroborated here by our whole-genome data (Supplementary Table 8). Conversely, the transcription factor ULTRAPETALA, which originated at the base of euphyllophytes and is present in *P.* *aquilinum*, was apparently secondarily lost in Salviniales (Supplementary Table 8). YABBY, an important transcription factor that patterns leaf polarity in flowering plants, is absent in our fern genomes and in the genome of the lycophyte *Selaginella moellendorffii*^15^ (Supplementary Table 8). Interestingly, a *YABBY* homologue was recently identified in a separate lycophyte species—*Huperzia selago*^16^—suggesting that *YABBY* has been lost at least twice in land plant evolution (in *Selaginella* and in ferns). How the differential retention of *YABBY* shaped the evolution of the vascular plant body plan requires further studies.

**Fig. 2:**
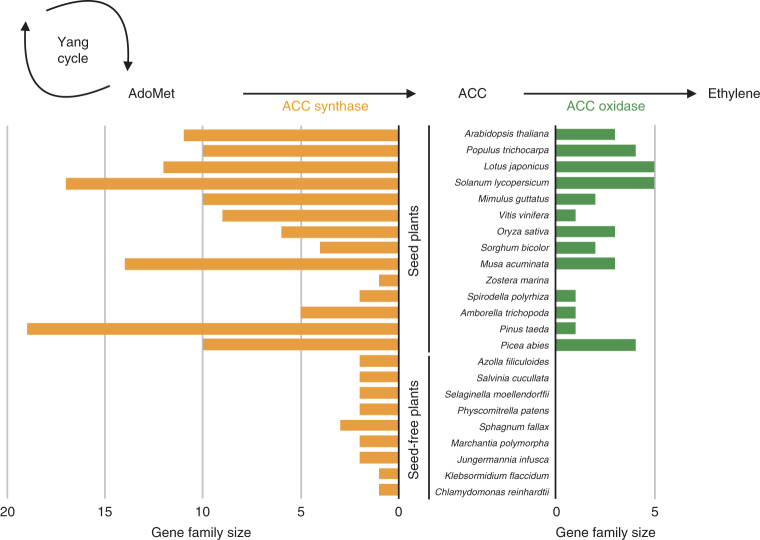
Evolution of ethylene biosynthesis. The ethylene-forming pathway involves the Yang cycle, where ACC is synthesized from *S*-adenosyl-methionine (SAM; also known as AdoMet) by ACC synthase. ACC oxidase then catalyses the conversion of ACC to ethylene. We found that ACC oxidase is unique to seed plants (green) and its origin probably drove the expansion of the ACC synthase gene family (orange; Supplementary Fig. 6) to create a regulated ethylene biosynthetic mechanism.

Among the orthogroups specific to seed plants, 1-aminocyclopropane-1-carboxylic acid (ACC) oxidase is of special interest because it converts ACC to ethylene—the last step in the ethylene biosynthetic pathway (Fig. 2). Ethylene is a critical plant hormone that controls various important physiological responses (for example, fruit ripening, flowering time, seed germination and internode elongation). Because ethylene responses are known in bryophytes, lycophytes and ferns^17^, it is intriguing to find that ACC oxidase only evolved within seed plants, a result confirming that seed-free plants must possess an alternative ethylene-forming mechanism^18^. Two other mechanisms, found in bacteria and fungi, result in ethylene formation: one via the 2-oxoglutarate-dependent ethylene-forming enzyme and the other through the non-enzymatic conversion of 2-keto-4-methylthiobutyric acid (KMBA) into ethylene^17^. We did not identify ethylene-forming enzyme in seed-free plant genomes, suggesting the absence of the ethylene-forming enzyme-based biosynthetic pathway. Seed-free plants may possibly synthesize ethylene non-enzymatically via KMBA; however, further biochemical studies are needed to test this hypothesis. Interestingly, ACC synthase (upstream of ACC oxidase) is present in seed-free plants, albeit in a lower copy number (<3) compared to seed plants, which average 9.3 copies (Fig. 2 and Supplementary Fig. 6). Paralogues of ACC synthase in seed plants are differentially regulated in response to varying developmental or environmental stimuli^19^. Thus, it is plausible that the expansion of the ACC synthase family was coupled with the origin of ACC oxidase in seed plants to create a regulated ethylene biosynthetic pathway.

### The history of whole-genome duplication in ferns

**Fig. 3:**
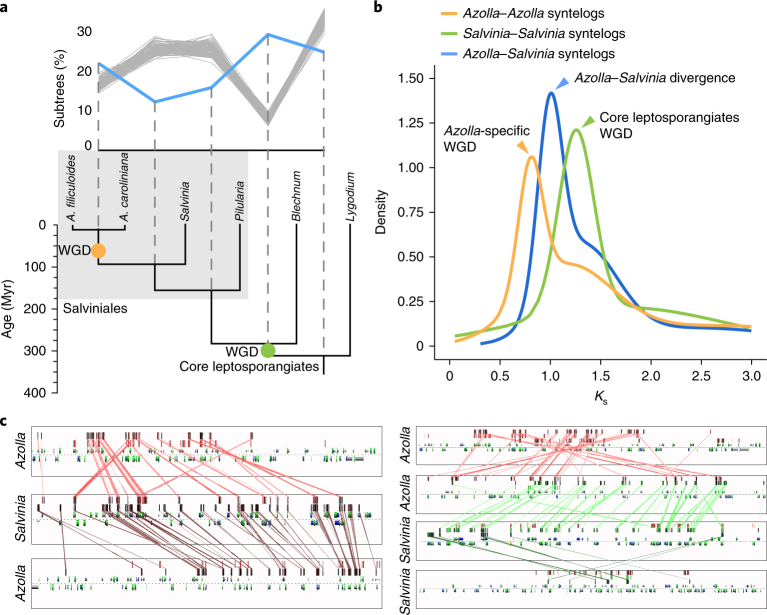
The history of WGD in *Azolla* and *Salvinia*. **a**, MAPS analysis identified two WGD events: one specific to *Azolla* (orange circle) and one predating the core leptosporangiates (green circle). The blue line illustrates the percentage of subtrees indicative of a gene duplication shared by the descendants at each node. The grey lines display the gene birth–death simulation results without WGD. The species divergence dates are from Testo and Sundue^129^. **b**, Density plots from fitting Gaussian mixture models to *K*_s_ distributions estimated from pairs of syntenic paralogues within the *Azolla* and *Salvinia* genomes, as well as of syntenic orthologues between *Azolla* and *Salvinia*. **c**, Examples of synteny between *Azolla* and *Salvinia* genomic regions. The left and right panels display a 2:1 and 2:2 syntenic relationship between *Azolla* and *Salvinia* regions, respectively. Each subpanel represents a genomic region in *Azolla* or *Salvinia*, with gene models on both strands shown above and below the dashed line. High-scoring sequence pairs (HSPs) in protein-coding sequences are marked by short vertical bars above the gene models. Selected HSP links between genomic regions are depicted as coloured lines crossing the subpanels, whereas others (for example, the HSP links between the two *Azolla* genomic regions in the left panel) are left out for clarity. Collinear series of HSPs across genomic regions indicates a syntenic relationship between the regions concerned. Genomic regions conserved in duplicate after the WGD that occurred prior to the divergence between *Azolla* and *Salvinia* should show a 2:2 syntenic relationship, whereas regions conserved in duplicate after the *Azolla*-specific WGD should show a 2:1 syntenic relationship with *Salvinia* regions. The left and right panels can be regenerated at https://genomevolution.org/r/ujll and https://genomevolution.org/r/ukys, respectively.

Our Multi-tAxon Paleopolyploidy Search (MAPS)^20^ phylogenomic analyses of the *Azolla* and *Salvinia* genomes (Fig. 3a), together with all available transcriptome data from other ferns, support two whole-genome duplication (WGD) events: a recent WGD event occurring in *Azolla* following its divergence from *Salvinia* and an earlier WGD predating the origin of ‘core leptosporangiates’ (sensu Pryer at al.^21^), a large clade comprising the heterosporous, tree and polypod ferns. The observed peaks of duplication associated with the inferred WGDs exceeded the 95% confidence intervals of our birth and death simulations for gene family evolution in the absence of WGDs. This high number of shared gene duplications is readily explained by a significant episodic birth event, such as a WGD. The discovery that *Azolla* experienced a genome duplication independent of other heterosporous ferns is not entirely surprising because *Azolla* has nearly twice the number of chromosomes of other heterosporous ferns, including *Salvinia* and *Pilularia*^22,23^ (Fig. 1a).

To further substantiate the two WGD events identified by MAPS, we examined the distribution of synonymous distances (*K*_s_) between syntenic paralogues within each of the genomes, as well as syntenic orthologues between *Azolla* and *Salvinia*. In the *Azolla* genome, we detected 242 syntenic blocks comprising 988 syntelog pairs. By contrast, only 83 syntenic blocks with 254 syntelog pairs could be found in *Salvinia*. Between *Azolla* and *Salvinia*, 3,587 pairs of syntenic orthologues were detected, clustering into 356 syntenic genomic blocks. We fit Gaussian mixture models to identify peaks in the *K*_s_ distributions (Fig. 3b and Supplementary Fig. 7). The main peak for *Azolla*–*Salvinia* orthologue pairs centres at ~1.0, which marks the species divergence between the two genera. To the left of this peak is the major *Azolla* intragenomic peak (~0.8), whose position confirms the *Azolla*-specific WGD event (Fig. 3b). To the right of the *Azolla*–*Salvinia* divergence peak is the *Salvinia* intragenomic *K*_s_ peak (~1.2–1.3), which matches a minor *Azolla* intragenomic peak, consistent with the proposed pre-core leptosporangiates WGD (Fig. 3b). Moreover, despite the antiquity of the WGDs and species divergence (Fig. 1a), we were still able to detect *Azolla*–*Salvinia* syntenic regions in a 2:1 or 2:2 syntenic relationship (Fig. 3c), respectively, corroborating the *Azolla*-specific and the older WGD events. The confirmation of these two WGDs in ferns further allows us to characterize patterns of gene retention following WGD. We found that *Azolla* syntenic paralogues are enriched for transcription-related genes (Supplementary Table 9), similar to what was observed in *Arabidopsis* and other angiosperms^24^. Likewise, protein kinases, another functional category commonly retained after WGD in seed plants, are significantly enriched in *Salvinia* syntenic paralogues (Supplementary Table 9). Additional genomic data are needed to better characterize the distribution of WGD events across the fern tree of life and to compare patterns of post-WGD gene fractionation with those documented in seed plants.

### The pentatricopeptide repeat family and RNA editing

The pentatricopeptide repeat (PPR) family is the largest gene family found in the *Azolla* and *Salvinia* genomes, with the *Azolla* genome encoding over 2,000 PPR proteins and the *Salvinia* genome over 1,700 PPR proteins. PPRs are implicated in organellar RNA processing^25^, and the large repertoire of PPRs correlates well with the extensive RNA editing we observed in the organellar genomes of Salviniales: 1,710 sites in *Azolla* organelles and 1,221 sites in *Salvinia* (Supplementary Table 10). These editing events include both C-to-U conversions (~70%) and U-to-C conversions (~30%). The number of PPR genes and the degree of RNA editing greatly exceed that found in seed plants and most bryophytes^26^. Of the sequenced plant genomes, only that of *S.* *moellendorffii*^15^ has more PPR genes^27^, correlating with the hyperediting seen in lycophytes^28^. However, there are no U-to-C editing events in *Selaginella*, making the *Azolla* and *Salvinia* genome sequences a novel and valuable resource for identifying the unknown factors catalysing these events.

More than half of the plastid transcripts and two-thirds of the mitochondrial transcripts in *Azolla* and *Salvinia* require start codon creation by C-to-U editing or stop codon removal by U-to-C editing before translation is possible. Most stop codon edits (76%) and start codon edits (62%) are shared between *Azolla* and *Salvinia* plastomes (as opposed to only 19% in internal ACG codons; Supplementary Fig. 10). This persistence of start and stop codon edits suggests that their loss is selected against, that is, creating the translatable sequence by RNA editing has an advantage over having it encoded by the genome. This argues that these particular RNA-editing events are not selectively neutral^29^ and supports editing as a control mechanism for gene expression in fern organelles.

Only ~55–60% of PPR proteins (1,220 in *Azolla* and 930 in *Salvinia*) contain domains associated with RNA editing in other plants. Although sufficient to account for the number of editing events observed (assuming each protein can specify one or a few sites as in other plants), this leaves a very large number of PPR proteins (~700 in *Azolla* and ~600 in *Salvinia*) with unknown functions. By comparison, flowering plants contain only 200–250 PPR proteins that lack editing domains.

### Origin and evolution of a fern insecticidal protein

**Fig. 4:**
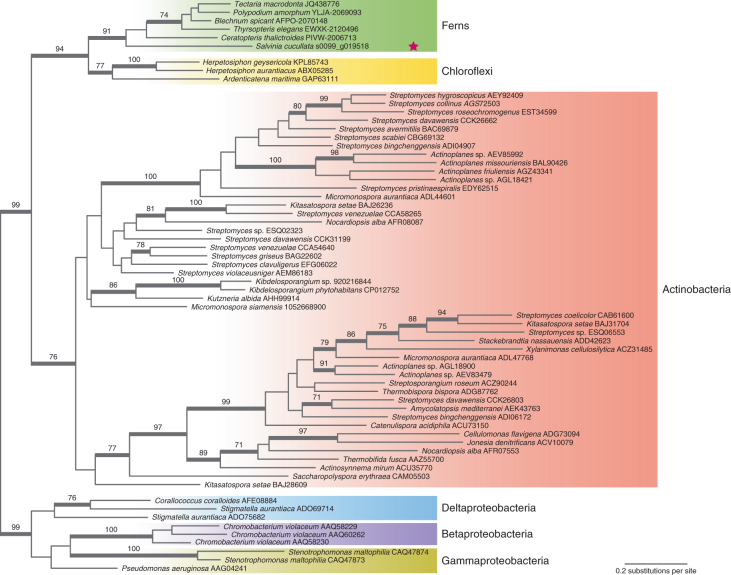
Origin of a fern insecticidal protein. Phylogenetic analysis of the chitin-binding domain Pfam PF03067 shows that the fern Tma12 insecticidal protein was probably derived from bacteria through an ancient HGT event. The numbers above the branches are bootstrap (BS) support values (BS = 100 is omitted), and the thickened branches indicate BS > 70. The tree is rooted based on the result from a broader phylogenetic analysis of PF03067 and PF08329 (Supplementary Data). The pink star denotes the sequence from the *S.* *cucullata* genome.

Ferns are remarkable for their high levels of insect resistance compared to flowering plants^30^. Recently, Shukla et al.^31^ isolated a novel insecticidal protein, Tma12, from the fern *Tectaria macrodonta*. Transgenic cottons carrying *Tma12* exhibit outstanding resistance to whitefly, yet show no decrease in yields, demonstrating tremendous agricultural potential. Tma12 has a high similarity to chitin-binding proteins (Pfam PF03067), but its evolutionary origin is unknown. Here, we found a *Tma12* homologue to be present in the *Salvinia* genome (henceforth *ScTma12*), as well as in a few 1,000 Plants (1KP)^32^ fern transcriptomes, but not in *Azolla* or any other publicly available plant genomes. Phylogenetic analyses position the fern *Tma12* sequences together with bacterial sequences, and are most closely related to the chitin-binding proteins from Chloroflexi (Fig. 4). We investigated whether this insecticidal protein was more likely a result of horizontal gene transfer (HGT) from bacteria to ferns or produced by fern-associated microorganisms. *ScTma12* is in a 646,687-bp scaffold (Sacu_v1.1_s0099) and has an 247-bp intron. The genes upstream and downstream of *ScTma12* are all clearly plant genes, and we found no abnormality in read-mapping quality, nor an abrupt change in read coverage (Supplementary Fig. 9), which together speak against the sequence being a contamination from a bacterial source. It has been argued that differential loss of genes in eukaryotes is the rule and gene acquisition by HGT rather rare^33^. The concerted loss of *Tma12* in each of the other Viridiplantae lineages is unlikely but cannot entirely be ruled out. However, functional HGT into eukaryotes does occur^34,35^ and *ScTma12* might represent such a case that contributed to the well-documented resistance of ferns against phytophagous insects.

### *Azolla*–cyanobacterial symbiosis

**Fig. 5:**
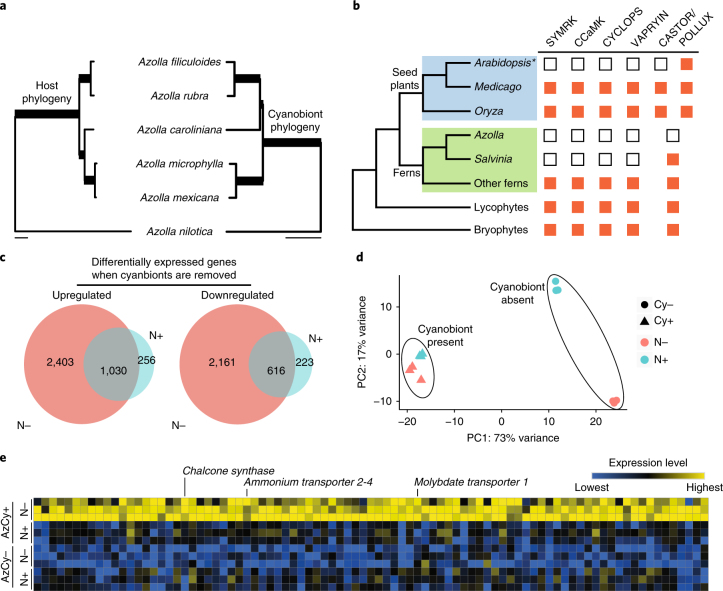
*Azolla*–cyanobacterial symbiosis. **a**, The cyanobiont phylogeny largely mirrors the host species phylogeny, indicating a convincing cospeciation pattern between the two partners. All nodes received a maximum likelihood bootstrap support of 100%, and for the host phylogeny, all nodes also received a local posterior probability of 1.0 from the ASTRAL^119^ analysis. Both the nuclear and the plastome data sets gave the same topology for the host, and the branch lengths shown here were from the plastome tree. Scale bars represent 0.01 substitutions per site. **b**, The CSP genes were lost in the *Azolla* and *Salvinia* genomes (empty boxes), whereas orthologues can be found in other fern transcriptomes (red boxes). *Arabidopsis lacks the CSP genes and does not have AM symbiosis. **c**, Cyanobionts have a large effect on the *Azolla* transcriptome. **d**, The *Azolla* transcriptome responds to nitrogen starvation more significantly when cyanobionts are absent than when they are present. PC, principal component. **e**, Candidate genes involved in nutrient transport and communication with cyanobionts.

To explore the co-evolutionary history of the *Azolla*–*Nostoc* symbiosis, we resequenced five other *Azolla* species and assembled each of their cyanobiont genomes. We then compared the cyanobiont phylogeny to the host species phylogeny and found a clear cospeciation pattern, with just one exception (the placement of *Azolla caroliniana*; Fig. 5a). Although such a pattern has been hinted at before^36,37^, we provide unequivocal evidence from whole-genome data. The genetic basis for this persistent symbiosis is undetermined. In plants, two other mutualistic associations—the arbuscular mycorrhizal (AM) and the nitrogen-fixing root nodule (RN) symbioses—have been well characterized. Whereas the AM symbiosis is formed between almost all land plants and a single fungal clade (Glomeromycota)^38^, the RN symbiosis is restricted to a few angiosperm lineages (mostly legumes) that associate with various nitrogen-fixing bacterial symbionts (for example, *Rhizobium* and *Frankia*). Despite these distinct differences, both symbioses require that a common symbiosis pathway (CSP) be established^38^. This pathway is highly conserved in all land plants^39^, except for those that have lost the AM symbiosis^40,41^, such as *A.* *thaliana* and three aquatic angiosperms^40,41^.

We investigated whether the CSP might have been co-opted during the evolution of the *Azolla*–*Nostoc* symbiosis by searching for six essential CSP genes in the *Azolla* and *Salvinia* genomes, as well as in transcriptomic data from other ferns in the 1KP data set^32^ (Supplementary Table 11). Although *DMI2* (also known as *SYMRK*), *DMI3* (also known as *CCaMK*), *IPD3* (also known as *CYCLOPS*) and *VAPYRIN* were found in other ferns, the *Azolla* and *Salvinia* genomes completely lacked orthologues (Fig. 5b). *IPD3* and *VAPYRIN* do not belong to multigene families^39^ and homologues were not detected. Although homologues of *DMI2* and *DMI3* were identified, phylogenetic analyses confirmed that they are not orthologous to the symbiotic genes (Supplementary Data). In addition, for *DMI3*, we searched the *Azolla* and *Salvinia* homologues for two motifs (threonine 271 and the calmodulin-binding domain) that are critical for symbiosis. Both motifs are missing from these sequences, confirming the absence of *DMI3*. *CASTOR* and *POLLUX* are paralogues resulting from a gene duplication event in the ancestor of seed plants, and although pre-duplicated homologues are present in *Salvinia* and other seed-free plants, they are absent in *Azolla* (Fig. 5b). The co-elimination of the CSP genes suggests the lack of AM symbiosis in *Azolla* and *Salvinia* and that the nitrogen-fixing *Azolla*–*Nostoc* symbiosis does not rely on this pathway.

To identify genes important for the *Azolla*–*Nostoc* symbiosis, we treated *A.* *filiculoides* with erythromycin to remove the cyanobiont (AzCy−) and compared its gene expression patterns with the wild type (AzCy+). Experiments were carried out in conditions where the nitrogen nutrient (ammonium nitrate) was either supplied (N+) or withheld (N−) from the growth media. Results from *nifH* real-time PCR confirmed the complete absence of cyanobacteria in AzCy− and showed that the addition of the nitrogen nutrient suppresses symbiotic N_2_ fixation in AzCy+ (Supplementary Fig. 10), consistent with an earlier study^42^. A large portion of the transcriptome is affected by the presence or absence of cyanobionts, with 6,210 and 2,125 genes being differentially transcribed under N− and N+ conditions, respectively (Fig. 5c and Supplementary Discussion). Of these, over 33% have at least a twofold expression difference. In response to nitrogen starvation, the *Azolla* transcriptomes remained moderately stable when the cyanobiont was present, but shifted drastically once it was absent (Fig. 5d). This finding suggests that the presence of the cyanobiont buffers the transcriptomic profile of *Azolla* from fluctuations in environmental nitrogen availability.

We focused primarily on those genes that are differentially expressed between the nitrogen treatments when the cyanobiont is present, and to a lesser extent on when the cyanobiont is absent (Fig. 5e and Supplementary Discussion). Because the cyanobacterial N_2_-fixation rate is strongly induced in the N− condition, we expect these genes to be candidates involved in nutrient exchange or in communication with the cyanobiont to promote N_2_ fixation. A total of 88 upregulated and 72 downregulated genes were identified (Fig. 5e). Among the upregulated genes is a paralogue of the ammonium transporter 2 subfamily (*AfAMT2-4*; Azfi_s0034.g025227; Fig. 5e and Supplementary Fig. 11) that is probably dedicated to ammonium uptake from the *Azolla* leaf cavity where the cyanobiont resides; homologous ammonium transporters have been implicated to participate in the AM and RN symbioses^43,44^. There is also a paralogue of the molybdate transporter gene family (*AfMOT1*; Azfi_s0167.g054529) that is most likely specialized for supplying molybdenum, a required co-factor for nitrogenase, to the cyanobiont. One of the legume *MOT1* genes was recently found to facilitate nitrogenase activity in RN symbiosis^45^. In addition to these two transporters, we identified a chalcone synthase paralogue in this candidate gene set. Chalcone synthase catalyses the production of naringenin chalcone and is the first committed step in flavonoid biosynthesis. Interestingly, naringenin and naringin both have significant effects on promoting cyanobacterial growth^46^ and differentiation^47^. Naringin is also a hormogonium-repressing factor^47^. Because hormogonia lack heterocysts and cannot fix nitrogen, naringin (or related flavonoids) could act as a plant signal to boost N_2_ fixation in the cyanobiont (Supplementary Discussion).

Although the ancient and intimate nature of the *Azolla*–*Nostoc* relationship suggests that gene transfer from *Nostoc* to the *Azolla* nuclear genome may have occurred over time, a thorough homology search found no evidence of *Nostoc*-to-*Azolla* HGT. However, we did discover a cyanobacteria-derived gene in the *Azolla* genome, but one that is shared with other ferns. This gene encodes a squalene–hopene cyclase (SHC), which mediates the cyclization of squalene into hopene, and is thought to be the evolutionary progenitor of many classes of eukaryotic and prokaryotic sterol cyclases. We found SHC homologues in both the *Azolla* and the *Salvinia* genomes, as well as in 40 fern 1KP transcriptomes. Our reconstructed gene phylogeny clearly shows that the fern SHCs are nested among cyanobacteria sequences (Supplementary Fig. 12). Although no homologue can be found in seed plants or in green algae, the SHC is also present in bryophyte (moss and liverwort) genomes and transcriptomes. Interestingly, these bryophyte SHCs are not related to those of ferns but are embedded in other bacterial SHC lineages (the monophyly of land plant SHCs is rejected by the Swofford–Olsen–Waddell–Hillis test^48^, *P* < 0.005). This finding implies a complex evolutionary history for SHCs in land plants, possibly featuring independent transfers of SHC from different prokaryotic lineages to mosses, liverworts and ferns. We are confident that these SHC genes are not from contaminants because the gene phylogeny largely mirrors the species phylogeny; furthermore, the SHC genes were not assembled into stray scaffolds in the genomes of *Azolla*, *Salvinia*, *Physcomitrella*^49^ or *Marchantia*^50^. In addition, we detected the triterpene products of SHC, hop-22(29)-ene, diplopterol and tetrahymanol, in *S.* *cucullata* biomass, providing direct evidence for SHC activity in this fern (Supplementary Fig. 13). Similar observations of SHC-synthesized triterpenes have been made in polypod ferns^51,52^ and mosses^53^. Because hopenes have an important role in plasma membrane stability in prokaryotes (similar to steroids in eukaryotes) and have been shown to confer low-temperature adaptation and stress tolerance^54^, it is plausible that the convergent evolution of hopene biosynthesis in seed-free plants, through independent HGTs from bacteria, might have contributed to the early adaptations of land plants to diverse and adverse environments. Functional studies are needed to confirm this hypothesis.

We anticipate that the availability of the first genomic data from ferns will continue to lead to vital insights into the processes that govern the evolution of plant genes and gene families. The implementation of fern data into the existing comparative genomic framework will enhance our understanding the plant tree of life.

## Methods

### Flow cytometry and genome size estimation

To estimate the genome sizes of *S.* *cucullata*, *P.* *americana*, *Regnellidium diphyllum* and *Marsilea minuta* (Supplementary Table 1), we used the Beckman chopping buffer to extract nuclei from fresh leaves, following the protocol of Kuo and Huang^55^. The nuclei extractions were mixed with those from standards, stained with 1/50 volume of propidium iodide solution (2.04 mg ml^−1^) and incubated at 4 °C in darkness for 1 h. For each species, three replicates were performed on the BD FACSCan system. For *S.* *cucullata*, we used *A.* *thaliana* (0.165 pg per C)^56^ as the standard, and for all other samples, we used *Zea mays* ‘CE-777’ (2.785 pg per C)^56^. For each peak (in both standard and sample), over 1,000 nuclei were collected with cross-validation values lower than 5%, except for those of *A.* *thaliana* 2*n* nuclei peaks, which ranged from 5.5% to 5.9%. To calculate the 2C-value of *S.* *cucullata*, we used a formula of: (0.66 pg × (*F* − S2*n*) + 0.33 pg × (S4*n* − *F*))/(S4*n* − S2*n*). For all other samples, we used: 5.57 pg × *F*/S2*n*, where 0.66, 0.33 and 5.57 pg are the 4C-value of *A.* *thaliana*, the 2C-value of *A.* *thaliana* and the 2C-value of *Z.* *mays* ‘CE-777’, respectively. S2*n*, S4*n* and *F* are the relative fluorescence amount (that is, the peak mean value) of the standard 2*n* nuclei, standard 4*n* nuclei and the sample 2*n* nuclei, respectively.

### Genome and transcriptome sequencing

*A.* *filiculoides* was collected from the Galgenwaard ditch in Utrecht, the Netherlands, and propagated directly or sterilized as described in Dijkhuizen et al.^57^. *A.* *filiculoides* (sterilized without cyanobiont) DNA was extracted, then sequenced on PacBio RSII at 51× coverage^57^ and Illumina HiSeq2000 (100 bp paired-end; ~86× coverage; Supplementary Table 12) with library insert sizes of 175 bp and 340 bp. RNA sequencing (RNA-seq) data from *A.* *filiculoides* of the Galgenwaard ditch used for annotation included the following RNA profiles: (1) at four time points during the diel cycle of fern sporophytes grown with or without 2 mM ammonium nitrate for 1 week^42^; (2) of different reproductive stages comparing fern sporophytes, microsporocarps and megasporocarps collected at noon^58^; (3) of roots treated with cytokinin, indole-3-acetic acid (IAA) or none^59^; and (4) of sporophytes with or without cyanobacterial symbionts grown with or without ammonium nitrate for 2 weeks then collected at noon. Plant materials of *A.* *caroliniana*, *Azolla mexicana*, *Azolla microphylla*, *Azolla nilotica* and *Azolla rubra* were obtained from the International Rice Research Institute (Supplementary Table 1) and DNA was extracted by a modified cetyltrimethylammonium bromide (CTAB) procedure^60^. Illumina libraries with a 500-bp insert size were prepared and sequenced on Illumina HiSeq2000 (100 bp paired-end; ~50× coverage; Supplementary Table 12).

*S.* *cucullata* was originally collected from Bangladesh and subsequently cultured at Taiwan Forestry Research Institute, Dr. Cecilia Koo Botanic Conservation Center and Duke University (Supplementary Table 1). Genomic DNA was purified using a modified CTAB procedure^60^ and sequenced on both PacBio RSII (10 SMRT cells; 46× coverage) and Illumina HiSeq2000 platforms (1 lane of 125 bp paired-end; 215× coverage; Supplementary Table 12). *S.* *cucullata* RNA from the floating and submerged leaves was separately extracted using the Sigma Spectrum Plant Total RNA kit, each with three biological replicates. To examine patterns of RNA editing, one library per leaf type was prepared by the Illumina Ribozero Plant kit (that is, not poly-A enriched), whereas the other two were done by the Kapa Stranded mRNA-seq kit. These six RNA libraries were pooled and sequenced in one lane of Illumina HiSeq2000 (125 bp paired-end).

### Genome assembly

We assembled the PacBio reads from *A.* *filiculoides* and *S.* *cucullata* genomes using PBcR^61^, and the resulting drafts were then polished by Quiver^62^ (*A.* *filiculoides*) or Pilon^63^ (*S.* *cucullata*). Plastid genomes were separately assembled using Mitobim^64^ and annotated in Geneious^65^ with manual adjustments. The PBcR contigs were filtered to remove plastome fragments. Although the *A.* *filiculoides* strain we sequenced was surface sterilized and treated with antibiotics to remove its associated microbiome, other endophytes could still persist, as shown by Dijkhuizen et al.^57^. Thus, we thoroughly assessed the *A.* *filiculoides* and *S.* *cucullata* assemblies to filter out all possible non-plant scaffolds. We used BlobTools^66^ in combination with SILVA^67^ and UniProt^68^ databases to infer the taxonomy for each scaffold. We removed all scaffolds that were classified as bacteria or fungi and also those that had a skewed GC content and read coverage. The completeness of each final assembly was assessed by BUSCO^69^ with the Plants set, and by using BWA^70^ and HISAT2^71^ to map Illumina reads to the assemblies (Supplementary Table 2).

### Repeat annotation

RepeatModeler^72^ was used to generate species-specific repeat libraries for masking and annotation. Consensus repeat sequences with homology to known plant genes were removed from the repeat libraries. Homology was defined as having a significant (E-value < 1 × 10^−5^) blastx^73^ hit to a subset of the PlantTribes^74^ v1.1 database that does not contain transposable element-related terms. Filtered RepeatModeler libraries were annotated with the name of the highest-scoring significant Repbase^75^ v22.04 full database sequence (tblastx^73^, E-value < 1 × 10^−5^) and the highest-scoring significant Dfam^76^ v2.0 profile hidden Markov model (HMM) (hmmsearch^77^, E-value < 1 × 10^−5^).

LTR-RTs were discovered using structural criteria by the GenomeTools^78^ program LTRHarvest^79^ with the following modifications to the default settings: a LTR similarity threshold of 0.01, an allowed LTR length range of 100–6,000 bp, an allowed distance between LTRs of a single element range of 1,000–25,000 bp and the number of bases outside LTR boundaries to search for target-site duplications set to 10. The GenomeTools program LTRDigest^80^ was used with a set of 138 transposable element-related Pfam profile HMMs to annotate protein-coding domains in the internal regions of LTR-RTs.

We used 38 previously published *A.* *filiculoides* RNA-seq libraries and 6 *S. cucullata* libraries (see above) to assemble transcriptomes for facilitating gene model predictions. Reads from *A.* *filiculoides* and *S.* *cucullata* libraries were processed using a combination of Scythe^81^ and Sickle^82^ or SOAPnuke^83^, with adapter and contaminating sequences discovered using FastQC^84^ (v0.11.5). Approximately 627 million (*A.* *filiculoides*) and 259 million (*S.* *cucullata*) cleaned paired reads went into the assemblies. Libraries from experimental replicates were combined and assembled de novo by Trinity^85^ (v2.0.6) and in a reference-guided manner using HISAT2^71^ (v2.0.4) and StringTie^86^ (v1.2.2), except for nine libraries published in de Vries et al.^59^ for which only a reference-guided approach was used. All programs used default parameters, and Trinity was run with the additional --trimmomatic option. StringTie results were merged using StringTie --merge, combined with the Trinity output, and were purged of redundant sequences using the GenomeTools sequniq utility^78^.

Putative centromere sequences were first identified by searching the genome assemblies with Tandem Repeat Finder^87^ to identify very high-copy (>100 repeats) tandem repeats with a motif length in the range of 185–195 bp. Motif sequences were extracted from the Tandem Repeat Finder output and clustered using USEARCH^88^. A single major cluster was identified for each species and the sequences were separately aligned using MAFFT^89^. Multiple sequence alignments for each species were used to generate a profile HMM representing the putative centromere sequences. We next used hmmsearch^77^ to search the genome assemblies again to identify all regions with similarity to the centromere profile HMMs. Genomic regions with significant HMM matches were identified and these regions were annotated in a GFF3 format.

### Gene prediction

Protein-coding genes were predicted using MAKER-P^90^ (v2.31.8), and three MAKER-P iterations were performed: (1) repeat masking and creation of initial gene models from transcript and homologous protein evidence; (2) refinement of initial models with SNAP^91^ ab initio gene predictor trained on initial models; and (3) final models generated using SNAP and the ab initio gene predictor AUGUSTUS^92^ trained on gene models from the second iteration.

Masking was performed by RepeatMasker^93^ (v4.0.5) using the previously described species-specific repeat libraries and the full Repbase v22.04 database. After masking, gene models were inferred from transcripts and homologous protein sequences by first aligning to the genomes using BLAST+^73^ (v2.2.31) blastn or blastp, and then refined using the functions est2genome and protein2genome from the splice-site aware alignment program Exonerate^94^ (v2.2.0). We included the previously described *A.* *filiculoides* or *S.* *cucullata* transcriptomes and the set of protein sequences consisting of the full Swiss-prot database (downloaded 18 June 2016), *Amborella trichopoda* v1.0 proteins, *A.* *thaliana* TAIR10 proteins, *Chlamydomonas reinhardtii* v5.5 proteins, *Oryza sativa* v7.0 proteins and *Physcomitrella patens* v3.3 proteins (from Phytozome^95^). Gene models with an annotation edit distance (AED) score of <0.2 were used to train SNAP, which was used during the second iteration of MAKER-P. SNAP was retrained for the final iteration using gene models from the second iteration with an AED score of <0.2 and a translated protein length of >200 amino acids. Prior to training AUGUSTUS^92^, redundant sequences, defined as those sharing ≥70% sequence similarity in significant (E-value < 1 × 10^−5^) HSPs from an all-by-all blastn alignment, were removed from the training set. Final non-redundant sets of 5,013 (*A.* *filiculoides*) or 6,475 (*S.* *cucullata*) gene models were used to train AUGUSTUS^92^.

### Phylogenomic inference and placement of WGDs from nuclear gene trees

To infer ancient WGDs, we used a gene-tree sorting and counting algorithm, implemented in the MAPS tool^20^. We selected four species of heterosporous ferns (two *Azolla*, one *Salvinia* and one *Pilularia*) and representatives of three other leptosporangiate lineages (*Blechnum*, *Lygodium* and *Dipteris*). The MAPS algorithm uses a given species tree to filter collections of nuclear gene trees for subtrees consistent with relationships at each node in the species tree. Using this filtered set of subtrees, MAPS identifies and records nodes with a gene duplication shared by descendant taxa. To infer and locate a potential WGD, we plotted the percentage of gene duplications shared by descendant taxa by node: a WGD will produce a large burst of shared duplications, appearing as an increase in the percentage of shared gene duplications^20^.

We circumscribed and constructed nuclear gene family phylogenies from multiple species for each MAPS analysis. We translated each transcriptome into amino acid sequences using the TransPipe pipeline^96^. Using these translations, we performed reciprocal protein BLAST (blastp) searches among data sets for each MAPS analysis using an E-value cut-off of 10^−5^. We clustered gene families from these BLAST results using OrthoFinder with the default parameters^97^ and only kept gene families that contained at least one gene copy from each taxon in a given MAPS analysis. We discarded the remaining OrthoFinder clusters. We used PASTA^98^ for automatic alignment and phylogenetic reconstruction of gene families, employing MAFFT^89^ for constructing alignments, MUSCLE^99^ for merging alignments and RAxML^100^ for tree estimation. The parameters for each software package were the default options for PASTA. For each gene family phylogeny, we ran PASTA until we reached three iterations without an improvement in the likelihood score using a centroid breaking strategy. We used the best-scoring PASTA tree for each multi-species nuclear gene family to infer and locate WGDs using MAPS.

For the null simulations, we first estimated the mean background gene duplication rate (*λ*) and the gene loss rate (*μ*) with WGDgc^101^. Gene count data were obtained from OrthoFinder clusters associated with each species tree. *λ* = 0.0031 and *μ* = 0.0039 were estimated using only gene clusters that spanned the root of their respective species trees, which has been shown to reduce biases in the maximum likelihood estimates of *λ* and *μ*^101^. We chose a maximum gene family size of 100 for parameter estimation, which was necessary to provide an upper bound for numerical integration of node states^101^. We provided a prior probability distribution of 1.5 on the number of genes at the root of each species tree, such that ancestral gene family sizes followed a shifted geometric distribution with a mean equal to the average number of genes per gene family across species.

Gene trees were then simulated within each MAPS species tree using the GuestTreeGen program from GenPhyloData^102^. We developed ultrametric species trees from the topological relationships inferred by the 1KP Consortium analyses and median branch lengths from TimeTree^103^. For each species tree, we simulated 4,000 gene trees with at least one tip per species: 1,000 gene trees at the *λ* and *μ* maximum likelihood estimates, 1,000 gene trees at half the estimated *λ* and *μ*, 1,000 trees at three times *λ* and *μ*, and 1,000 trees at five times *λ* and *μ*.

### Classification of syntenic duplicates and microsynteny analysis

To distinguish gene duplicates as syntenic or tandem, we used the SynMap^104^ tool from the CoGe^105^ platform, with default parameters and the Quota Align algorithm to merge syntenic blocks. Sets of syntenic paralogues or orthologues (defined by a collinear series of putative homologous genes) were extracted using the DAGChainer algorithm, whereas duplicates within ten genes apart in the same genomic region were identified as tandem duplicates (Supplementary Discussion). Results for within *Azolla* and *Salvinia* genome comparisons, as well as between *Azolla* and *Salvinia*, can be regenerated using the links https://genomevolution.org/r/tozk, https://genomevolution.org/r/toz7 and https://genomevolution.org/r/toyy, respectively. Microsynteny analyses were performed using the GEvo tool from CoGe^105^. We used the default setting to define the minimum number of collinear genes for two regions to be called syntenic. Non-coding regions were masked in the two genomes to include only the protein-coding sequences. The two example microsyntenies shown in Fig. 5c can be regenerated at https://genomevolution.org/r/ujll and https://genomevolution.org/r/ukys.

### Gaussian mixture model analysis of *K*_s_ distributions

Estimates of *K*_s_ were obtained for all pairs of syntenic paralogous and orthologous genes using the CODEML program^106^ in the PAML package (v4.8)^107^ on the basis of codon sequence alignments. We used the GY model with stationary codon frequencies empirically estimated by the F3 × 4 model. Codon sequences were aligned with PRANK (version 100701) using the empirical codon model^108^ (setting -codon) to align coding DNA, always skipping insertions (-F). Only gene pairs with *K*_s_ values in the range of 0.05–5 were considered for further analyses. Gaussian mixture models were fitted to the resulting frequency distributions of *K*_s_ values by means of the densityMclust function in the R mclust version 5.3 package^109^. The Bayesian information criterion was used to determine the best-fitting model for the data, including the optimal number of Gaussian components to a maximum of nine. For each component, several parameters were computed including the mean and the variance, as well as the density mixing probabilities and the total number of gene pairs.

### Gene family classification and ancestral reconstruction

The OrthoFinder^97^ clustering method was used to classify complete proteomes of 23 sequenced green plant genomes, including *A.* *filiculoides* and *S.* *cucullata* (Supplementary Table 5), into orthologous gene lineages (that is, orthogroups). We selected taxa that represented all of the major land plant and green algal lineages, including six core eudicots (*A.* *thaliana*, *Lotus japonicus*, *Populus trichocarpa*, *Solanum lycopersicum*, *Erythranthe guttata* and *Vitis vinifera*), five monocots (*O.* *sativa*, *Sorghum bicolor*, *Musa acuminata*, *Zostera marina* and *Spirodella polyrhiza*), one basal angiosperm (*A.* *trichopoda*), two gymnosperms (*Pinus taeda* and *Picea abies*), two ferns (*A.* *filiculoides* and *S.* *cucullata*), one lycophyte (*S.* *moellendorffii*), four bryophytes (*Sphagnum fallax*, *P.* *patens*, *Marchantia polymorpha* and *Jungermannia infusca*) and two green algae (*Klebsormidium flaccidum* and *C.* *reinhardtii*). In total, 16,817 orthogroups containing at least two genes were circumscribed, 8,680 of which contain at least one gene from either *A.* *filiculoides* or *S.* *cucullata*. Of the 20,203 annotated *A.* *filiculoides* genes and the 19,780 annotated *S.* *cucullata* genes, 17,941 (89%) and 16,807 (84%) were classified into orthogroups, respectively. The details for each orthogroup, including gene counts, secondary clustering of orthogroups (that is, super-orthogroups)^110^ and functional annotations, are reported in Supplementary Table 5.

We used Wagner parsimony implemented in the program Count^111^ with a weighted gene gain penalty of 1.2 to reconstruct the ancestral gene content at key nodes in the phylogeny of the 23 land plants and green algae species (Supplementary Table 5). The ancestral gene content dynamics—gains, losses, expansions and contractions—are depicted in Supplementary Fig. 5. Complete details of orthogroup dynamics for the key ancestral nodes that include seed plants, such as Salviniaceae, euphyllophytes and vascular plants, are reported in Supplementary Table 5.

### Transcription-associated protein characterization

Transcription-associated proteins comprise transcription factors that bind in a sequence-specific manner to *cis*-regulatory DNA elements and transcriptional regulators that act via protein–protein interaction or chromatin modification. We conducted genome-wide, domain-based annotation of transcription-associated proteins according to previous studies^14,112^. A total of 1,206 (6%, *Azolla*) and 983 (7%, *Salvinia*) proteins were sorted into families; this amount is similar to *Selaginella* but less than in gymnosperms or angiosperms (Supplementary Table 8).

### PPR annotation

We conducted a targeted annotation for PPR genes because they are generally only weakly expressed and thus often lack transcriptome support. Open reading frames from the nuclear genome assemblies were translated into amino acid sequences using the “getorf” tool from the EMBOSS (v.6.5.7) package^113^ with a minimum size restriction of 300 nucleotides. These open reading frames were searched for PPR motifs using the hmmsearch tool from the HMMER3 package^77^. The PPR motif models and parameters used follow those of Cheng et al.^27^. Motifs were assembled into full PPR tracts and the best model for each PPR was determined^27^.

To study the prevalence and location of RNA editing, non-poly(A)-enriched RNA-seq data were filtered to remove adapters, low-quality reads and reads with ≥5% Ns. Clean reads were aligned against the assembled plastid and mitochondrial genome assemblies using TopHat 2.0 (ref.^114^). One of the inverted repeat regions in the plastid genomes was removed before mapping. Only uniquely mapped reads were retained as input for SAMtools^115^ to call mismatches between RNA and the corresponding DNA. Differences between corresponding RNA and DNA sequences were identified as the putative RNA-editing sites. The RNA-editing level was defined as the number of altered reads divided by the total mapped reads for each site.

### Phylogeny of the insecticidal protein Tma12

We used BLASTp^73^ to search for *Tma12* (Genbank accession: JQ438776) homologues in Phytozome^95^, 1KP transcriptomes^32^ and the NCBI Genbank non-redundant protein database. Although *Tma12* homologues are present in fern transcriptomes and in the *S.* *cucullata* genome, no significant hit was found in any other plant genomes or transcriptomes. In addition, the majority of the Tma12 protein is composed of a chitin-binding domain that belongs to the PF03067 Pfam family. This family does not contain any plant genes but is predominantly represented in the genomes of Actinobacteria, insects and fungi. To trace the origin of fern *Tma12* genes, we downloaded representative sequences containing PF03067 and PF08329 (as the outgroup) from UniProt and Genbank and reconstructed the phylogeny using IQ-TREE^116^. We then used this preliminary phylogeny (Supplementary Data) to construct a more focused data set to narrow down the phylogenetic affinity of *Tma12*. PartitionFinder^117^ was used to infer the optimal codon partition scheme and substitution models, and RAxML^100^ was used for maximum likelihood phylogeny inference and to calculate bootstrap branch support.

### *Azolla* phylogeny

From the resequencing data (Supplementary Table 12), we compiled both plastome and nuclear phylogenomic data sets to infer the *Azolla* species phylogeny. *S.* *cucullata* was used as the outgroup. For the plastome phylogeny, we concatenated nucleotide alignments from 83 protein-coding genes and used PartitionFinder^117^ to identify the optimal data partition scheme and the associated nucleotide substitution models. RAxML^100^ was used for maximum likelihood phylogeny inference and to calculate bootstrap branch support. For the nuclear data set, we focused on genes that, based on the gene family classification results, are single copy in both *A.* *filiculoides* and *S.* *cucullata* genomes. We used HybPiper^118^ to extract the exon sequences from each of the resequenced species. The ‘bwa’ option was used in HybPiper instead of the ‘blastx’ default. We then filtered out genes with more than two species missing or having an average sequence length shorter than 75% of the one in *A.* *filiculoides*. This resulted in a final data set of 2,108 genes. Sequence alignments and gene tree inferences were done in PASTA^98^, with the default setting, except that RAxML^100^ was used to estimate the best tree on the final alignment. To infer the species tree from these gene trees, we used the multi-species coalescent method implemented in ASTRAL-III (v5.6.1)^119^. The tree topology from the plastome and nuclear data sets were identical, and all nodes received bootstrap support of 100 and a local posterior probability of 1.0.

### Cyanobiont phylogeny

To compare the host and symbiont phylogenies, we assembled the cyanobiont genomes from five additional *Azolla* species (Supplementary Table 12) using the resequencing data generated from total DNAs, including sequences derived from both the host and the cyanobiont. To extract the cyanobiont genomes from each of the *Azolla* species, we first filtered out chloroplast sequences by using BWA^70^ (default parameters) to map the total clean DNA reads against each chloroplast genome reference. In this step, ~3–4% of the reads were filtered out, which is necessary to remove plastid ribosomal RNAs that are highly similar to ones in the cyanobionts. For each of the five *Azolla* species, we then mapped the filtered reads to the published cyanobiont reference (*N.* *azollae* 0708 isolated from *A.* *filiculoides*^11^; Genbank accession: NC_014248) using BLAST^73^ (alignment criteria: E-value ≤ 1 × 10^−5^, sequence identity of ≥90% and an aligned coverage of ≥90%). Only the aligned reads were assembled by Mitobim^64^ (iterations = 5) using *N.* *azollae* 0708 (ref.^11^) as a reference. Gene prediction for each assembled cyanobiont was performed by the Prodigal program^120^. Transfer RNAs were predicted by tRNAscan-SE^121^ using a bacterial tRNA gene structure model. The presence of rRNA sequences (gene number and structure) for each cyanobiont was confirmed by mapping the rRNAs of *N.* *azollae* 0708 against each assembled genome cyanobiont sequence using BLAST. We used mugsy^122^ to generate the whole-genome alignment, which resulted in a nucleotide matrix of 5,354,840 characters. IQ-TREE^116^ was used for model testing and maximum likelihood tree inference. Because the *N.* *azollae* genome is reduced in size and is significantly diverged from other cyanobacteria, we could not find an appropriate outgroup to root the cyanobiont tree. To overcome this, we used STRIDE^123^ implemented in OrthoFinder^97^ to locate the root by reconciling gene trees. STRIDE was run with the default setting, except that MAFFT^89^ was used for alignment and RAxML^100^ for tree inference. The root was found to be the node placing the *A.* *nilotica* cyanobiont as sister to a clade comprising all other cyanobionts. The reconciled species tree is identical to the tree reconstructed from the whole-genome alignment.

### Identification of the CSP genes

The *Medicago truncatula DMI2*, *DMI3*, *IPD3*, *CASTER/POLLUX* and *VAPYRIN* sequences were used as queries, as in a previous study^39^, to search against the genomes and transcriptomes from species listed in Supplementary Table 11 using tBLASTn^73^. For liverworts and ferns from the 1KP data set^32^, non-annotated transcriptomes were used as targets, with the longest open reading frame of each contig extracted and translated. For *A.* *filiculoides* and *S.* *cucullata*, both the annotated gene models and the unannotated scaffolds were used. All hits that matched already annotated gene models were discarded prior to subsequent analyses. No homologues were identified in the two fern genomes for *IPD3* and *VAPYRIN*. Protein sequences for *DMI2*/*SYMRK*, *DMI3*/*CCaMK* and *CASTOR*/*POLLUX* were aligned using MAFFT^89^. The best substitution model for each alignment (JTT for all alignments) was determined using MEGA6 (ref.^124^). Phylogenetic trees were generated using RAxML^100^ on the CIPRES platform^125^, and node support was assessed with 100 rapid bootstrap pseudoreplicates.

### Quantitative real-time PCR of *nifH*

Quantitative real-time PCR for the *N.* *azollae nifH* gene was conducted using total RNA extracted from *A.* *filiculoides*. Primers were derived from Brouwer et al.^58^. ThermoFisher Superscript IV was used to generate complementary DNA from the RNA. The cDNA was then used for quantitative PCR with the Roche SYBR Green Master Mix on a Chromo4 real-time PCR machine with the Opticon platform. The relative gene expression was calculated using the 2^ΔC(t)^ method, with the cyanobacteria present/nitrogen absent condition as the reference.

### *Azolla* symbiosis transcriptome analysis

We used RNA-seq to compare gene expression patterns of AzCy+ and AzCy− individuals grown with or without ammonium nitrate. Each condition and treatment combination has three biological replicates. RNA-seq reads were mapped to the *A.* *filiculoides* genome by HISAT2^71^, and read counts for each gene were calculated using the HTSeq software package^126^. We used the rlog function in the DESeq2 package^127^ for data normalization and carried out differential expression analysis in DESeq2 to identify upregulated and downregulated genes with an adjusted *P* value of 0.005. Distance clustering and principal component analysis were used to examine the relatedness of samples and conditions as a quality-control measure.

### *Azolla*–cyanobacteria HGT

To identify cyanobiont-derived genes in the *A.* *filiculoides* genome, we first investigated a potential orthologous relationship between any *Azolla* genes and cyanobacteria. For this, we used the *Azolla* genome assembly as a query for a DIAMOND BLASTx^128^ against a protein data set of 11 cyanobacterial genomes. This resulted in 30,312 *Azolla* genome contigs hitting 8,779 different cyanobacterial proteins that were used as a query in a tBLASTn^73^ against the *Azolla* genome; 340 *Azolla* contigs had reciprocal hits. To investigate whether these represent possible *Nostoc*-to-*Azolla* transfers or just examples of plastid-to-nucleus transfers, we used the 340 *Azolla* contigs for another BLASTx against the cyanobacteria and extracted all 51,743 BLASTx*-*aligned *Azolla* sequences. These highly redundant protein sequences were used for a DIAMOND BLASTp against the non-redundant database of NCBI. Almost all of the sequences had streptophyte proteins as the top hit, and when not, phylogenetic analysis clearly placed them within streptophytes.

### Phylogeny of SHC

Homologues of SHC and oxidosqualene cyclase were obtained by searching against Phytozome^95^, 1KP transcriptomes^32^ and the NCBI Genbank non-redundant protein database. Protein alignment was done in MUSCLE^99^. We used IQ-TREE^116^ to find the best-fitting amino acid substitution model and infer the phylogeny using maximum likelihood. Bootstrap support was assessed with 1,000 pseudoreplicates. To test whether the monophyly of fern, lycophyte, moss and liverwort SHC could be rejected, we conducted a Swofford–Olsen–Waddell–Hillis test using SOWHAT^48^. We compared the best maximum likelihood topology against the topology with all land plant SHC constrained to be monophyletic. SOWHAT was run with 1,000 replicates.

### Detection of SHC-synthesized triterpenes

Freeze-dried *S.* *cucullata* biomass was Soxhlet extracted in a 9:1 DCM:MeOH mixture for 24 h. The total lipid extracts obtained were dried over Na_2_SO_4_ followed by evaporation of the solvent by a gentle stream of N_2_. Aliquots of the total lipid extracts were methylated with diazomethane to convert the acid groups into corresponding methyl esters, purified over a SiO_2_ column and silylated using bis(trimethylsilyl)trifluoracetamide (BSTFA) in pyridine at 60 °C for 20 min to convert the hydroxy groups into the corresponding trimethylsilyl ethers. The total lipid extracts were on-column injected on a Thermo Trace GC Ultra Trace DSQ gas chromatography mass spectrometry (GC–MS) onto a CP-sil 5CB-fused silica column (30 m × 0.32 mm internal diameter, film thickness: 0.10 μm). The GC–MS was operated at a constant flow of 1.0 ml min^−1^. The GC oven was programmed starting at 70 °C to rise to 130 °C at a rate of 20 °C per min and then to 320 °C at a rate of 4 °C per min, followed by an isothermal hold for 20 min.

### Reporting Summary

Further information on experimental design is available in the Nature Research Reporting Summary linked to this article.

### Data availability

The genome assemblies and annotations can be found at www.fernbase.org. The raw genomic and transcriptomic reads generated in this study were deposited in the NCBI SRA under the BioProject PRJNA430527 and PRJNA430459. The sequence alignments and tree files can be found in the Supplementary Data.

## Supplementary information

Supplementary Information Supplementary Discussion, Supplementary References, Supplementary Figures 1–13, Supplementary Tables 1–4 and Supplementary Data.

Reporting Summary

Supplementary Table 5 Gene family classification and dynamics.

Supplementary Table 6 Evolution of transcription factors involved in seed development.

Supplementary Table 7 Transcription factors gained and expanded in seed plants.

Supplementary Table 8 Annotations of transcription associated proteins (TAP).

Supplementary Table 9 Gene ontology terms enriched in syntenic paralogues and tandem duplicates.

Supplementary Table 10 Summary of RNA-editing in *Azolla filiculoides* and *Salvinia cucullata* organellar genomes.

Supplementary Table 11 Annotations of common symbiosis genes.

Supplementary Table 12 Summary of the sequence data generated in this study.
