# Supplementary material for: Fern genomes elucidate land plant evolution and cyanobacterial symbioses
Source: Nat Plants. 2018 Jul 2;4(7):460–72. doi: 10.1038/s41477-018-0188-8 (PMC6786969; doi:10.1038/s41477-018-0188-8)
Supplement: Supplementary file 1 — Supplementary Discussion, Supplementary References, Supplementary Figures 1–13, Supplementary Tables 1–4 and Supplementary Data. [file 41477_2018_188_MOESM1_ESM.pdf]

nature plants

SUPPLEMENTARY INFORMATION
https://doi.org/10.1038/s41477-018-0188-8

In the format provided by the authors and unedited.

Fern genomes elucidate land plant evolution and cyanobacterial symbioses

Fay-Wei Li[1,2]*, Paul Brouwer[3], Lorenzo Carretero-Paulet[4,5], Shifeng Cheng[6], Jan de Vries[7], Pierre-Marc Delaux[8], Ariana Eily[9], Nils Koppers[10], Li-Yaung Kuo[1], Zheng Li[11], Mathew Simenc[12], Ian Small[13], Eric Wafula[14], Stephany Angarita[12], Michael S. Barker[11], Andrea Bräutigam[15], Claude dePamphilis[14], Sven Gould[16], Prashant S. Hosmani[1], Yao-Moan Huang[17], Bruno Huettel[18], Yoichiro Kato[19], Xin Liu[6], Steven Maere[4,5], Rose McDowell[13], Lukas A. Mueller[1], Klaas G. J. Nierop[20], Stefan A. Rensing[21], Tanner Robison[22], Carl J. Rothfels[23], Erin M. Sigel[24], Yue Song[6], Prakash R. Timilsena[14], Yves Van de Peer[4,5,25], Hongli Wang[6], Per K. I. Wilhelmsson[21], Paul G. Wolf[22], Xun Xu[6], Joshua P. Der[12], Henriette Schluepmann[3], Gane K.-S. Wong[6,26] and Kathleen M. Pryer[9]

[1]Boyce Thompson Institute, Ithaca, NY, USA. [2]Plant Biology Section, Cornell University, Ithaca, NY, USA. [3]Molecular Plant Physiology Department, Utrecht University, Utrecht, the Netherlands. [4]Bioinformatics Institute Ghent and Department of Plant Biotechnology and Bioinformatics, Ghent University, Ghent, Belgium. [5]VIB Center for Plant Systems Biology, Ghent, Belgium. [6]BGI-Shenzhen, Beishan Industrial Zone, Shenzhen, China. [7]Department of Biochemistry and Molecular Biology, Dalhousie University, Halifax, Nova Scotia, Canada. [8]Laboratoire de Recherche en Sciences Végétales, Université de Toulouse, CNRS, UPS, Castanet Tolosan, France. [9]Department of Biology, Duke University, Durham, NC, USA. [10]Department of Plant Biochemistry, Cluster of Excellence on Plant Sciences, Heinrich Heine University Düsseldorf, Dusseldorf, Germany. [11]Department of Ecology and Evolutionary Biology, University of Arizona, Tucson, AZ, USA. [12]Department of Biological Science, California State University, Fullerton, CA, USA. [13]ARC Centre of Excellence in Plant Energy Biology, School of Molecular Sciences, The University of Western Australia, Crawley, Western Australia, Australia. [14]Department of Biology, Huck Institutes of the Life Sciences, Pennsylvania State University, University Park, PA, USA. [15]Faculty of Biology, Bielefeld University, Bielefeld, Germany. [16]Institute for Molecular Evolution, Heinrich Heine University Düsseldorf, Dusseldorf, Germany. [17]Taiwan Forestry Research Institute, Taipei, Taiwan. [18]Max Planck Genome Centre Cologne, Max Planck Institute for Plant Breeding, Cologne, Germany. [19]Institute for Sustainable Agro-ecosystem Services, University of Tokyo, Tokyo, Japan. [20]Geolab, Faculty of Geosciences, Utrecht University, Utrecht, the Netherlands. [21]Faculty of Biology, University of Marburg, Marburg, Germany. [22]Department of Biology, Utah State University, Logan, UT, USA. [23]University Herbarium and Department of Integrative Biology, University of California, Berkeley, CA, USA. [24]Department of Biology, University of Louisiana, Lafayette, LA, USA. [25]Department of Biochemistry, Genetics and Microbiology, University of Pretoria, Pretoria, South Africa. [26]Department of Biological Sciences, Department of Medicine, University of Alberta, Edmonton, Alberta, Canada. *e-mail: fl329@cornell.edu

Supplementary Information

- **Supplementary Discussion**
  - **Genome annotation**
  - **Tandem gene duplications**
  - ***Azolla*-cyanobacteria symbiosis**
- **Supplementary Figure 1.** *Azolla filiculoides* and *Salvinia cucullata* genome annotations.
- **Supplementary Figure 2.** Density of genes and repeats.
- **Supplementary Figure 3.** Genomic distribution of tRNA genes.
- **Supplementary Figure 4.** Divergence estimates of LTR retrotransposons.
- **Supplementary Figure 5.** Ancestral gene family reconstruction.
- **Supplementary Figure 6.** Phylogeny of ACC synthase (ACS).
- **Supplementary Figure 7.** Distributions of Ks values of syntenic paralogs and syntenic orthologs.
- **Supplementary Figure 8.** RNA-editing in *Azolla filiculoides* and *Salvinia cucullata* plastomes.
- **Supplementary Figure 9.** *ScTma12* is a nuclear-encoded gene in *Salvinia cucullata* genome.
- **Supplementary Figure 10.** Cyanobacterial *NifH* expressions in *Azolla filiculoides*.
- **Supplementary Figure 11.** Gene expression patterns of selected transporter genes.
- **Supplementary Figure 12.** Phylogeny of squalene-hopene cyclase (SHC).
- **Supplementary Figure 13.** Identification of SHC-synthesized triterpenes in *Salvinia cucullata*.
- **Supplementary Table 1**. Voucher table.
- **Supplementary Table 2**. Genome assembly statistics.
- **Supplementary Table 3.** Gene annotation statistics.
- **Supplementary Table 4.** Repeat annotation results.
- **Supplementary Table 5.** Gene family classification and dynamics. (as a separate excel file)
- **Supplementary Table 6.** Evolution of transcription factors involved in seed development. (as a separate excel file)
- **Supplementary Table 7.** Transcription factors gained and expanded in seed plants. (as a separate excel file)
- **Supplementary Table 8.** Annotations of transcription associated proteins (TAP). (as a separate excel file)
- **Supplementary Table 9.** Gene ontology terms enriched in syntenic paralogs and tandem duplicates. (as a separate excel file)
- **Supplementary Table 10.** Summary of RNA-editing in *Azolla filiculoides* and *Salvinia cucullata* organellar genomes. (as a separate word file)
- **Supplementary Table 11.** Annotations of common symbiosis genes. (as a separate excel file)
- **Supplementary Table 12.** Summary of the sequence data generated in this study. (as a separate excel file)

Supplementary Data. Sequence alignments and tree files of:

- **ACC synthase**
- **Tma12**
- ***Azolla* nuclear phylogeny**
- ***Azolla* plastome phylogeny**
- **Azolla cyanobiont phylogeny**
- **Common symbiosis genes**
- **Squalene hopene cyclase**

Supplementary Discussion

Genome annotation

Gene annotation

We identified 51,098 and 28,968 protein-coding gene models in *Azolla* and *Salvinia*, respectively, using the MAKER-P pipeline[1] (Supplementary Fig. 2). Genes were classified as high-confidence (HC) if they were supported by transcript evidence or had significant sequence similarity to other known plant proteins (Supplementary Fig. 1, Supplementary Table 3). Gene models only supported by *ab initio* predictions were classified as low-confidence (LC) and were excluded from analyses of gene families. The mean length of HC protein-coding genes is 5 kb and 3.4 kb with a mean of 5.3 and 5.2 introns per gene in *Azolla* and *Salvinia*, respectively (Supplementary Table 3).

RNA gene profiles

The number of rRNA genes is similar in *Azolla* and *Salvinia* (1,397 and 1,161, respectively; Supplementary Fig. 2, Supplementary Table 3). In contrast, the *Salvinia* genome contains 50% more tRNA genes (an increase of 3,515 genes) compared to *Azolla.* These tRNA genes are primarily distributed evenly across the genome in both species (Supplementary Fig. 3), but a few tRNA genes appear to have proliferated locally. For example, high numbers of tRNA-Glu genes are clustered on scaffolds 43, 46, and 48 in *Salvinia*, and tRNA-Asp genes are clustered on scaffolds 10 and 19 in *Azolla* (Supplementary Fig. 3). *Azolla* has nearly twice as many tRNA-Asp genes as its second most abundant tRNA, 95% of which have one (ATC) of the two possible Asp anticodons. The two most abundant tRNA gene types in *Salvinia* are tRNA-Arg and tRNA-Glu, which are 4.5 and 6.3 times more than the third (tRNA-His). Like *Azolla,* specific anticodons are disproportionately represented (Supplementary Fig. 3).

Repetitive elements

In *Azolla*, we found 17,484 putative full length long terminal repeat retrotransposons (LTR-RT), more than six times the number in *Salvinia* (Supplementary Figs. 1 and 4). We estimated sequence divergences between LTRs for all full length LTR-RT predictions that were supported by having homology to LTR-RTs in the Dfam 2.0 and Repbase 22.04 databases. Assuming a low rate of gene conversion among LTRs[2], the divergence between LTRs could serve as a proxy for time since element insertion due to the nature of the LTR-RT transposition mechanism. Interestingly, the density plots in Supplementary Fig. 4 show the distribution of LTR divergences in *Salvinia* as potentially bimodal. Given a constant background mutation rate and a constant birth rate for LTR-RTs, one would expect a smoothly tapered right-skewed distribution. The bimodality could be due to recent deletion of many newer LTR-RTs, a burst of transposition in the past, and/or heterogeneous historical substitution rates. The *Azolla* and *Salvinia* assemblies include 12.138 Mb and 13.095 Mb of centromere-like sequences, respectively. These sequences are concentrated on particular scaffolds and have been identified on 514 scaffolds in *Azolla* and 940 scaffolds in *Salvinia* (Supplementary Fig. 2).

Tandem gene duplications

In addition to examining gene evolution associated with whole genome duplications, we also characterized tandem gene duplication in the *Azolla* and *Salvinia* genomes. To distinguish gene duplicates as syntenic or tandem, we used SynMap and *DAGChainer* algorithm to extract syntenic paralogs. Duplicates that are within ten genes apart in the same region of the genome were identified as tandem duplicates. Functional enrichment analysis revealed the GO term 'protein binding' as the most significantly over-represented in both *Azolla* and *Salvinia* tandemly duplicated genes, most of them annotated as belonging to the highly diverged pentatricopeptide repeat protein (PPR) family. A second group of *Azolla* tandem duplicates was found to be involved in chitin-binding and chitinase activities, belonging to a distinct family of glycosyl hydrolases involved in breaking down glycoside bonds in chitin, a polymer of the glucose derivative N-acetylglucosamine found in the cell walls of fungi and the exoskeletons of arthropods such as crustaceans and insects. These tandem genes formed a cluster of 12 genes, located in a genomic region syntenic to a cluster of four tandem duplicates in the *Salvinia* genome (the microsynteny analysis can be regenerated at https://genomevolution.org/r/zsy2).

Azolla-cyanobacteria symbiosis

Global gene expression pattern comparing cyano-absent and cyano-present individuals

A total of 6,644 genes are differentially expressed between AzCy- and AzCy+ individuals, and 2,254 of them exhibit at least 2-fold expression difference. Under the N- conditions, 3,433 genes are up-regulated and 2,777 are down-regulated. Far fewer genes, 1,286 and 839 genes, are respectively up- and down-regulated under the N+ conditions.

Candidate gene set

We show here that cyanobacterial N_2-fixation rate is highly induced when plants are grown without nitrogen nutrient (Supplementary Fig. 10), indicating an active control of plants on the cyanobionts. To identify likely candidates involved in this symbiotic regulation, we focused on genes that, when cyanobionts are present, are differentially expressed between the N treatments, but not or to a lesser degree when cyanobionts are gone. In other words, for the up-regulated genes, they have to satisfy these three criteria: (1) when cyanobionts are present, they have a higher expression in N- than in N+, (2) when limited by nitrogen nutrients, they have a higher expression in cyano+ than cyano-, and (3) they are not down-regulated in N- compared to N+ when cyano-. And the opposite pattern would apply to the down-regulated genes. We found a total of 88 up-regulated and 72 down-regulated genes in this category that we termed "putative symbiotic genes". These include an ammonium transporter, a metal ion transporter, and a chalcone synthase that might be involved in flavonoid signaling. The importance of these genes is discussed below.

Symbiosis-specific transporters

Azolla has five ammonium transporter paralogs (*AMT*) within its genome. Ammonium transporters come in two major classes in plants: *AMT1*s and *AMT2*s. In plants, *AMT1* genes are mainly expressed in the roots, and are responsible for transporting ammonium from the external environment into the xylem. These genes are usually constitutively expressed. In contrast,

*AMT2*s are inducibly-expressed in all other plant tissues, such as shoots, leaves, and flowers. *Azolla filiculoides* has one *AMT1* (Azfi_s0034.g025388) and four *AMT2*s. One *A. filiculoides AMT2*, *AfAMT2-4* (Azfi_s0034.g025227), appears to be symbiosis-specific, as its expression is up-regulated when the cyanbiont is present, particularly under the nitrogen-depleted condition (i.e. when cyanobionts are fixing the most nitrogen; Supplementary Fig. 11). *AfAMT2-4* is therefore likely the main transporter for exchange of ammonium with *Nostoc* in the leaf pocket. On the other hand, the expression profile of *AfAMT2-3* (Azfi_s0093.g043301) suggests that it is a nitrogen-starvation responsive gene, whereas *AfAMT1* is likely a general ammonium transporter, as it is expressed similarly regardless of cyanobacterial presence (Supplementary Fig. 11). The *AfAMT2-1* and *AfAMT2-2* genes are nearly identical to each other, so that their expressions cannot be measured correctly and were thus excluded. In addition to ammonium, there are myriad cofactors that are needed by *Nostoc* for N_2-fixation. Metal ions, such as molybdenum, copper, and iron, are among the most crucial of these cofactors[3,4]. We found a particular paralog of molybdate transporter (*AfMOT1*; Azfi_s0167.g054529) and a paralog of vacuolar iron transporter (*AfVIT*) in the putative symbiotic gene list (Supplementary Fig. 11). Similarly, in *Medicago truncatula*, a root nodule-specific *MtMOT1.3* paralog was recently identified to mediate Mo transfer from plants to the symbiotic rhizobium[5].

Possible roles of flavonoids in Azolla-cyanobacteria communication

The identification of a chalcone synthase (CHS) in our putative symbiosis gene list is of particular interest (Figure 5e). CHS produces naringenin chalcone, and is the first committed step in flavonoid biosynthesis pathway. Flavonoids are major plant signals used in symbioses with rhizobia and *Frankia*. Silencing of CHS in *Medicago truncatula*[6] and *Casuarina glauca*[7] both resulted in a defective nodule formation. Interestingly, flavonoids also have significant effects on cyanobacteria growth and cellular differentiation. Naringenin was shown to stimulate growth of a number of cyanobacteria species including ones in *Nostoc*[8]. Furthermore, naringin was found to be one of the most potent hormogonia-repressing factors (HRF)[9]. Hormogonia are the motile stage of cyanobacteria and do not contain N_2-fixing heterocysts. In the *Azolla-Nostoc* symbiosis, hormogonia are maintained in the shoot apex, and upon entering nascent leaf cavities, they return to the vegetative stage, and develop heterocysts for N_2-fixation. Because hormogonia cannot fix nitrogen, boosting the hormogonia-repressing signals can promote N_2-fixation rates and cyanobacteria maturation. Given the expression pattern of CHS, we hypothesize that flavonoids act as a HRF in *Azolla-Nostoc* symbiosis, and are a major communication signal to help time the development of the leaf cavity with the metabolic development of the cyanobiont. Consistent with our hypothesis, *Azolla* aqueous extract was found to contain flavonoids and, importantly, can effectively suppress hormogonia differentiation[10].

References

1. Campbell, M. S. *et al.* MAKER-P: a tool kit for the rapid creation, management, and quality control of plant genome annotations. *Plant Physiol.* **164,** 513–524 (2014).
2. Cossu, R. M. *et al.* LTR retrotransposons show low levels of unequal recombination and high rates of intraelement gene conversion in large plant genomes. *Genome Biol. Evol.* **9,** 3449–3462 (2017).
3. González-Guerrero, M., Matthiadis, A., Sáez, Á. & Long, T. A. Fixating on metals: new insights into the role of metals in nodulation and symbiotic nitrogen fixation. *Front. Plant Sci.* **5,** 45 (2014).
4. Brear, E. M., Day, D. A. & Smith, P. M. C. Iron: an essential micronutrient for the legume-rhizobium symbiosis. *Front. Plant Sci.* **4,** 359 (2013).
5. Tejada-Jiménez, M. *et al. Medicago truncatula* Molybdate Transporter type 1 (MtMOT1.3) is a plasma membrane molybdenum transporter required for nitrogenase activity in root nodules under molybdenum deficiency. *New Phytol* **216,** 1223–1235 (2017).
6. Wasson, A. P., Pellerone, F. I. & Mathesius, U. Silencing the flavonoid pathway in *Medicago truncatula* inhibits root nodule formation and prevents auxin transport regulation by rhizobia. *Plant Cell* **18,** 1617–1629 (2006).
7. Abdel-Lateif, K. *et al.* Silencing of the chalcone synthase gene in *Casuarina glauca* highlights the important role of flavonoids during nodulation. *New Phytol* **199,** 1012–1021 (2013).
8. Żyszka, B., Anioł, M. & Lipok, J. Modulation of the growth and metabolic response of cyanobacteria by the multifaceted activity of naringenin. *PLoS ONE* **12,** e0177631 (2017).
9. Cohen, M. F. & Yamasaki, H. Flavonoid-induced expression of a symbiosis-related gene in the cyanobacterium *Nostoc punctiforme*. *J. Bacteriol.* **182,** 4644–4646 (2000).
10. Cohen, M. F., Sakihama, Y., Takagi, Y. C., Ichiba, T. & Yamasaki, H. Synergistic effect of deoxyanthocyanins from symbiotic fern *Azolla* spp. on hrmA gene induction in the cyanobacterium *Nostoc punctiforme*. *Mol. Plant Microbe Interact.* **15,** 875–882 (2002).

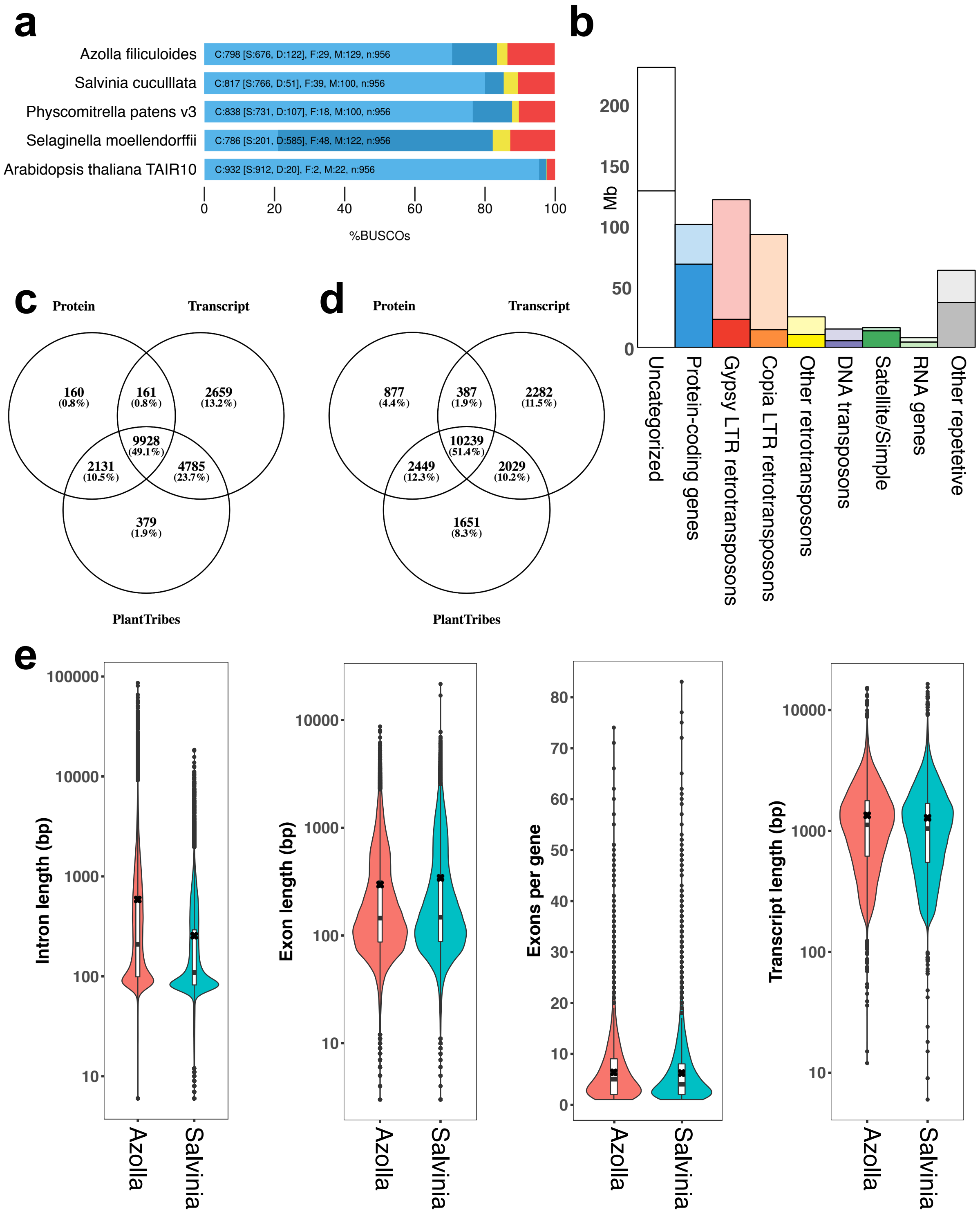

Supplementary Figure 1. Summary of *Azolla filiculoides* and *Salvinia cucullata* genome annotations. (a) Comparison of BUSCO scores (the Plants set) of *Azolla* and *Salvinia* assemblies with other sequenced plant genomes. Blue, yellow, and red bars respectively illustrate the proportion of complete (C), fragmented (F), and missing (M) BUSCO genes; the dark blue bar is for complete but duplicated BUSCO genes. (b) The annotated genomic compositions, with *Salvinia* as lower bars (vibrant colors) and *Azolla* as upper bars (diffuse colors). Identification of high-confidence (HC) protein-coding genes in (c) *Azolla* and (d) *Salvinia*. HC genes were identified as having evidence from RNA-seq data, or similarity to protein data in UniProt/SwissProt, *Selaginella*, *Chlamydomonas*, *Arabidopsis*, *Oryza*, *Amborella*, or the PlantTribes 22 Genomes v1.1 database. (e) Distribution of HC gene features: intron length, exon length, number of exons per gene, and transcript length in *Azolla* (red) and *Salvinia* (blue).

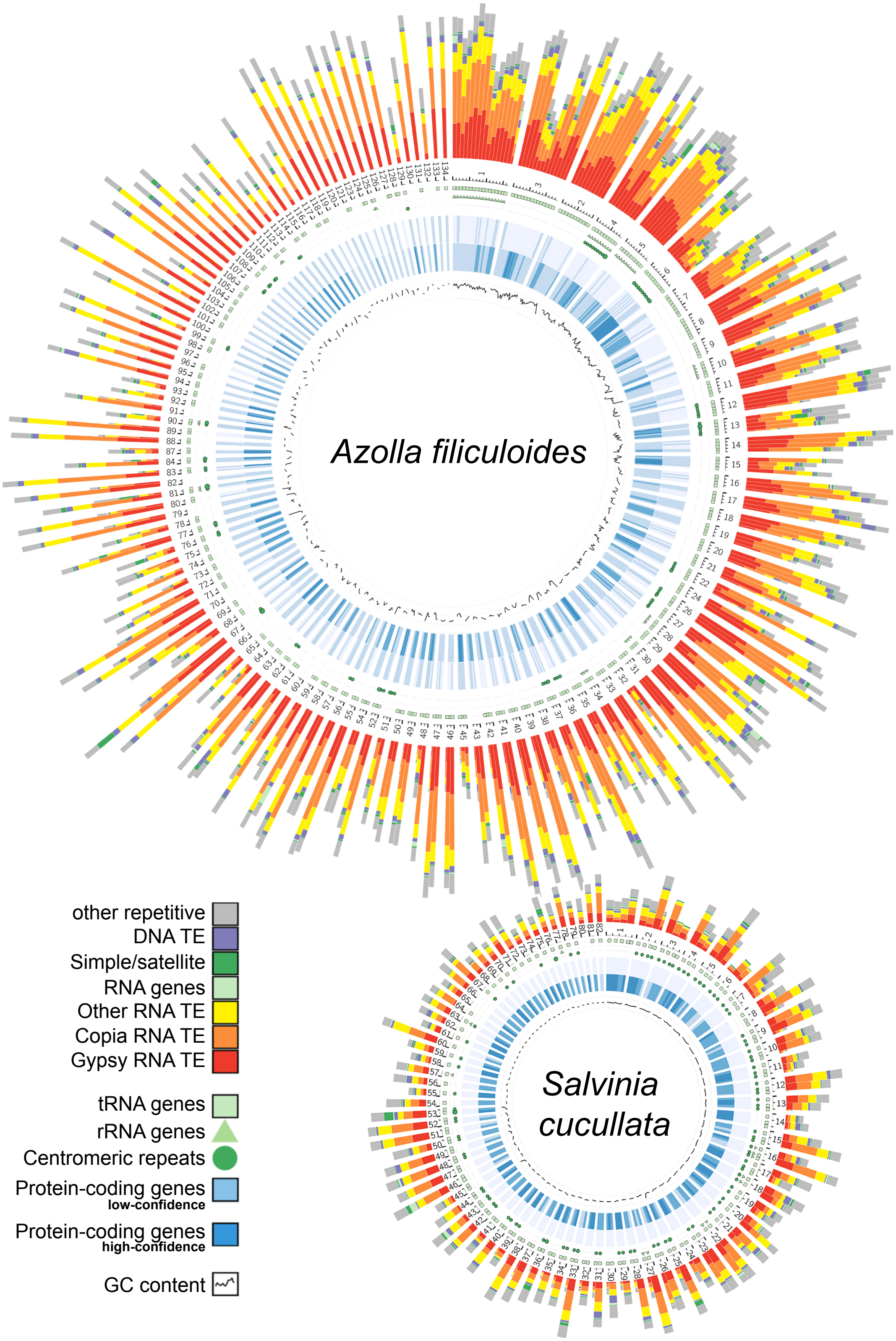

Supplementary Figure 2. Circos plots showing density of genes and repeats across the largest scaffolds comprising half of the *Azolla filiculoides* and *Salvinia cucullata* genome assemblies. Circular plot areas are proportional to the amount of sequence shown.

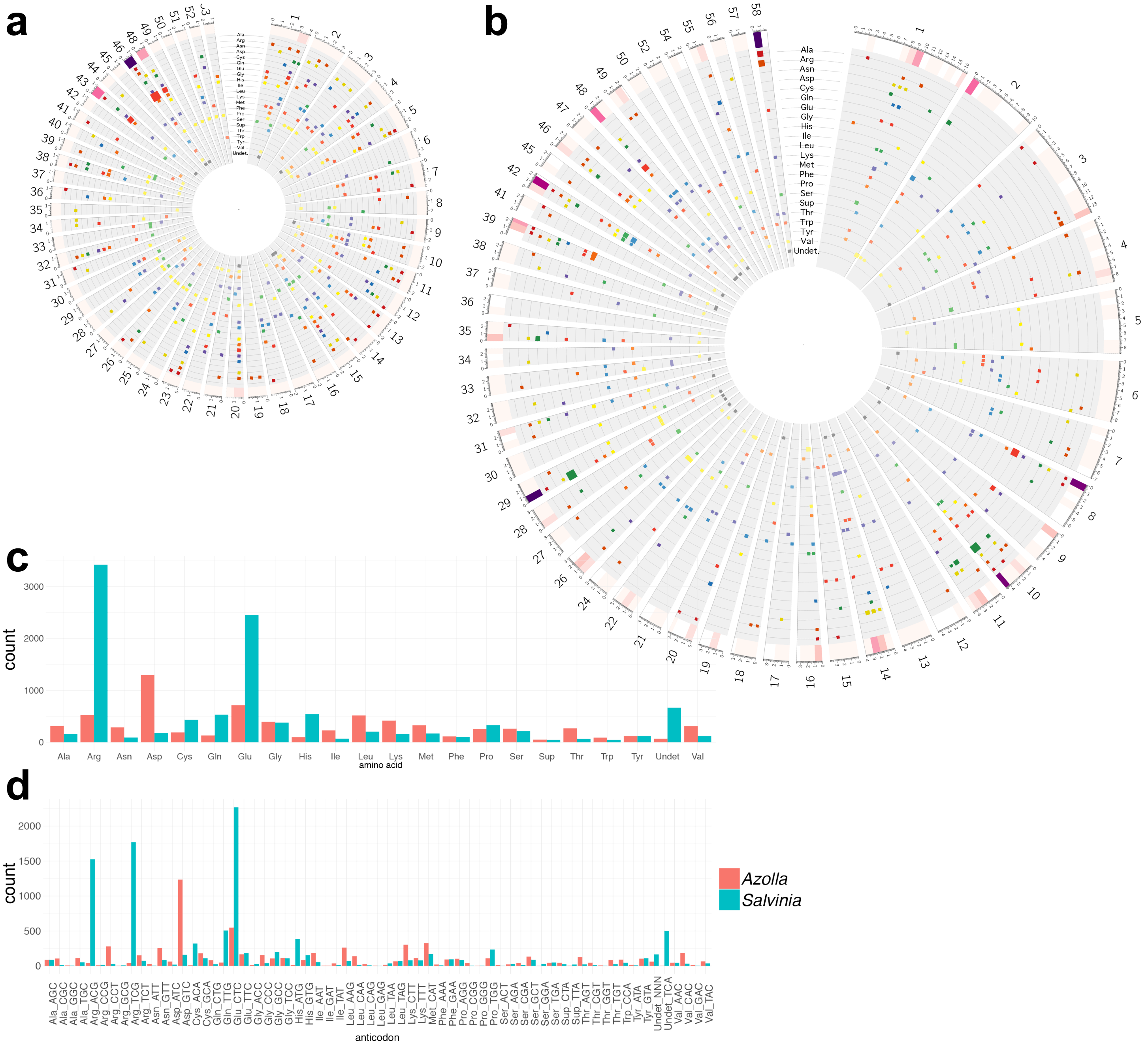

Supplementary Figure 3. Genomic distribution of tRNA genes. Circos plots showing locations and densities of non-pseudogenized tRNA genes predicted by tRNAscan-SE organized by their predicted anticodon for the largest scaffolds greater than 1 Mb for *Salvinia* (a) and *Azolla* (b). The shade of each 1 Mb region in the outermost track corresponds to the total tRNA density for that sequence region. Each inner track shows the location of each 1 Mb sequence region that contains tRNA genes for a specific amino acid; square size is proportional to the density of the given tRNA gene in that region. Numbers of tRNA genes in genome by amino acid (c) and anticodon (d). Circular plot areas are proportional to the amount of sequence shown.

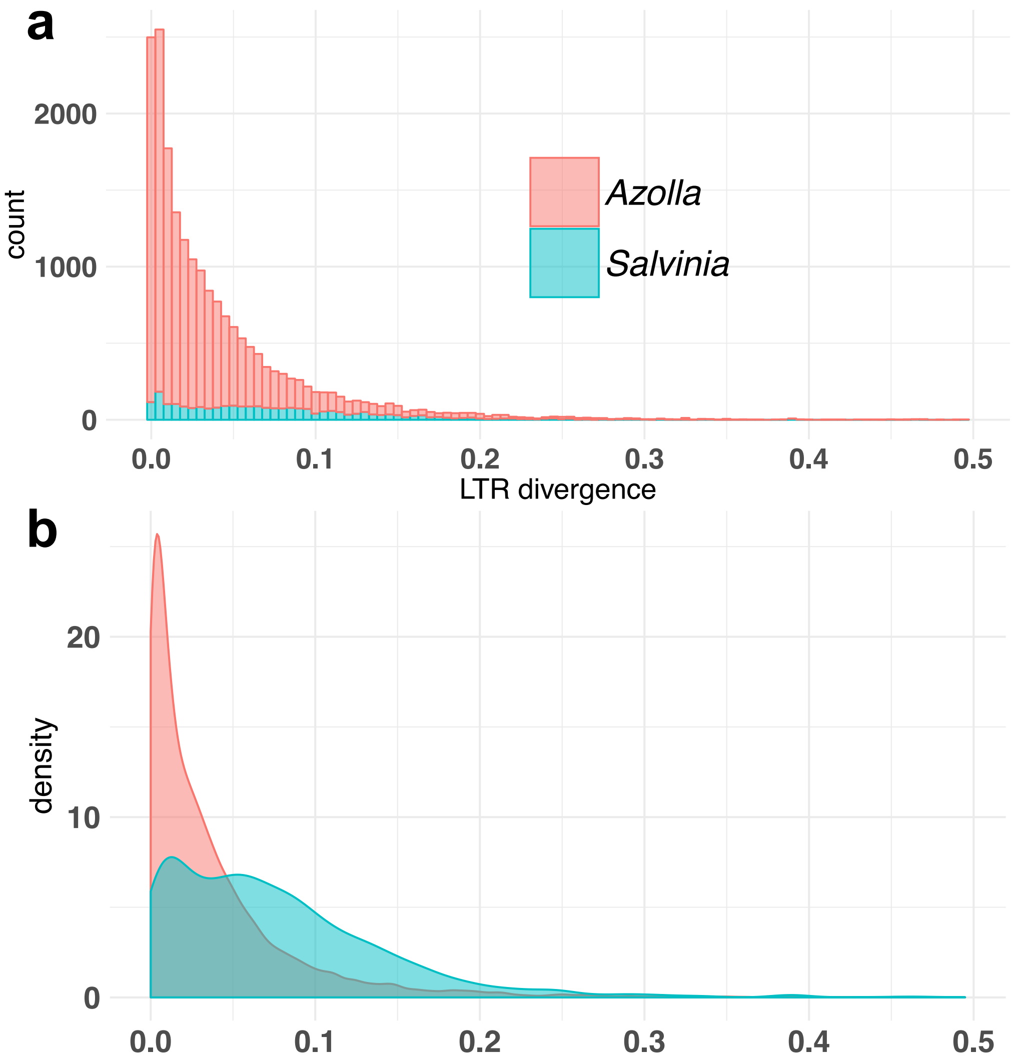

Supplementary Figure 4. Density plots (a) and histograms (b) of divergence estimates for long terminal repeat (LTR) pairs of 17,286 LTR retrotransposons in *Azolla* and 2,526 in *Salvinia*.

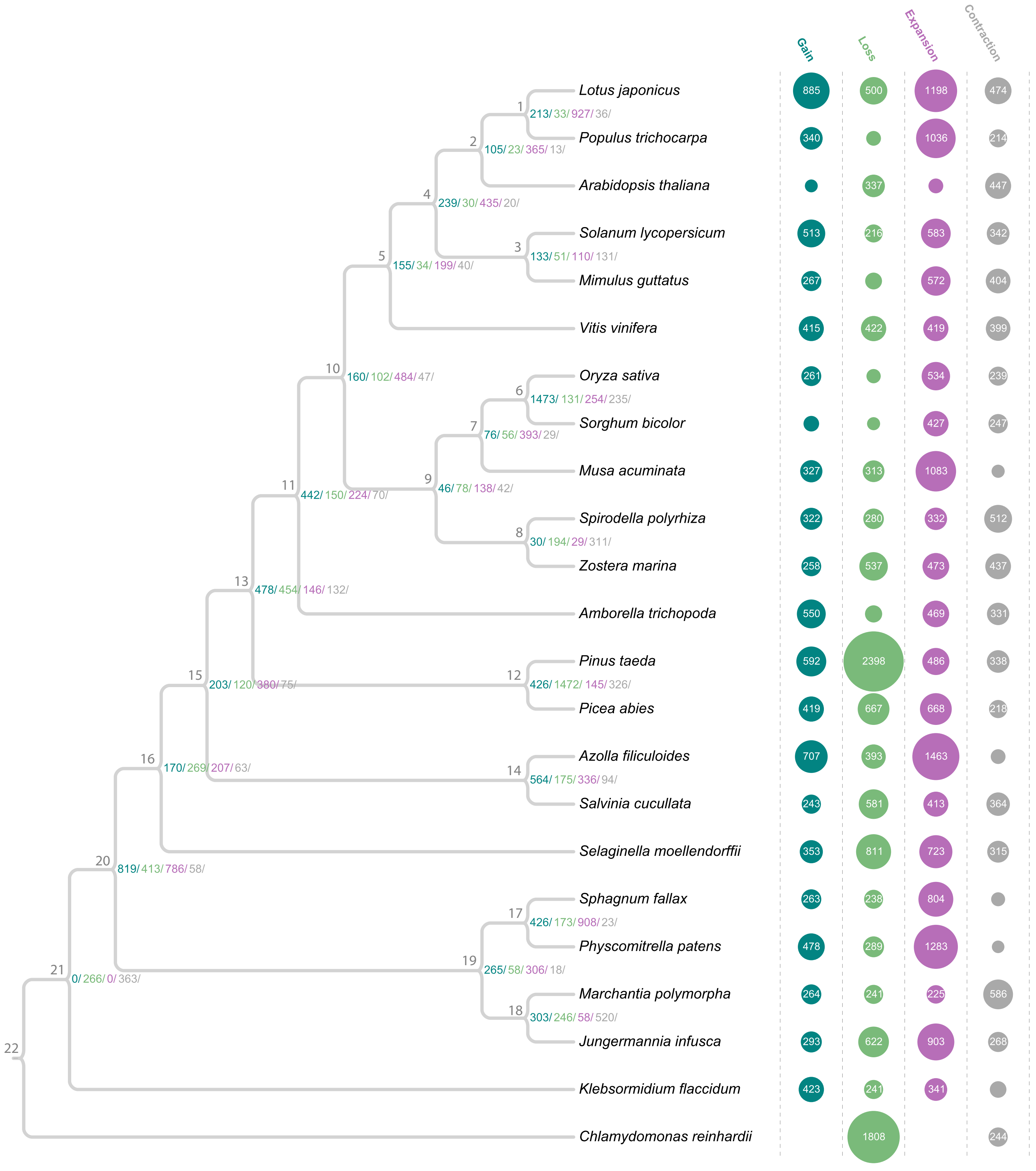

Supplementary Figure 5. Ancestral gene family reconstruction inferred from the global gene family classification of proteins from 22 land plants and 2 green algae genomes. Evolutionary events are mapped on each internal node of the species phylogeny representing orthogroups gains (teal), losses (green), expansions (magenta), and contractions (gray). Circles with inset numbers represent the terminal nodes with the size proportional to the number of inferred orthogroups.

Supplementary Figure 6. Phylogeny of ACC synthase (ACS). Seed plants have an expanded ACS repertoire compared to seed -free plants. The numbers above branches are bootstrap (BS) support values (BS=100 is omitted), and the thickened branches indicate BS>70.

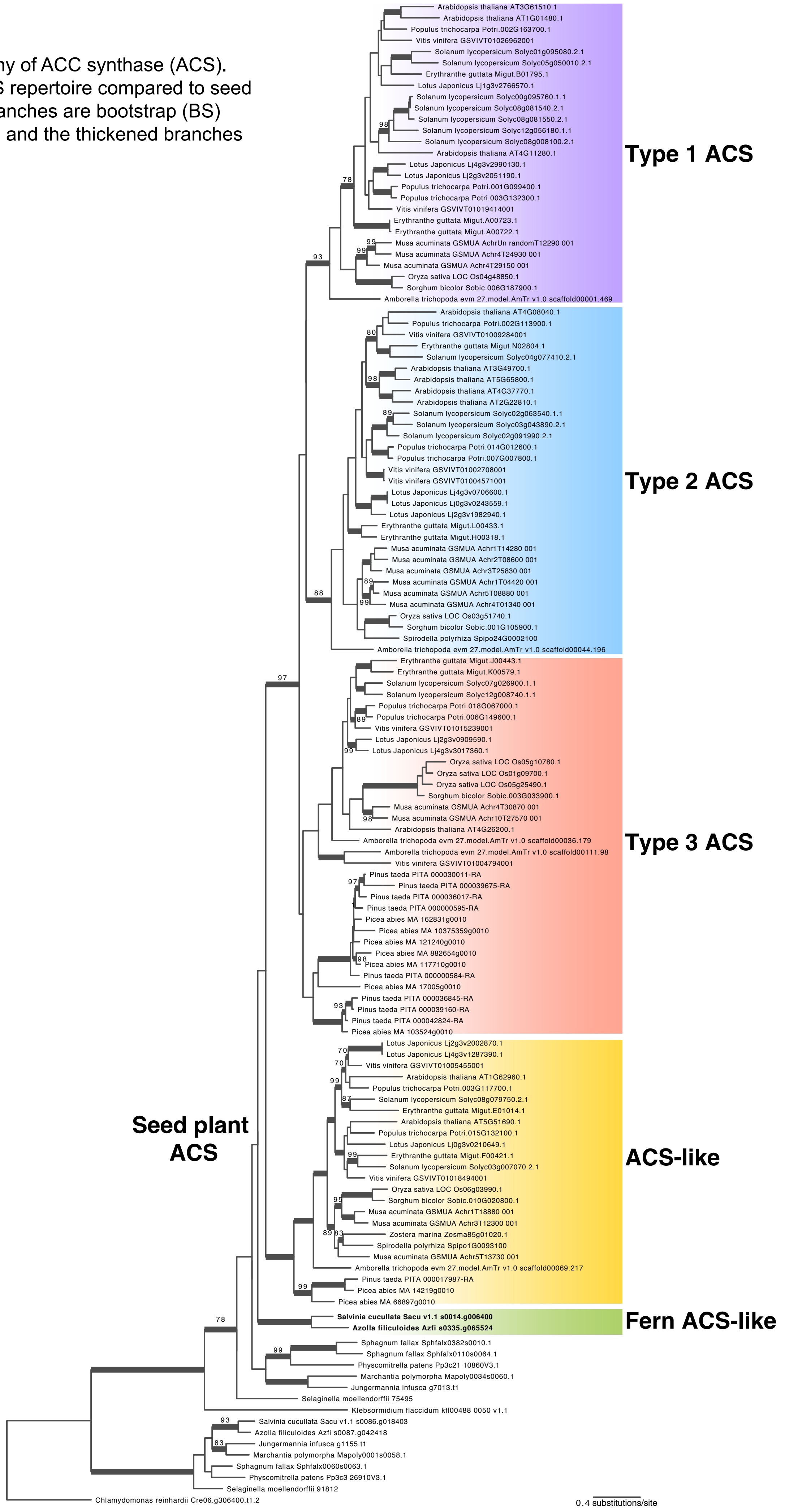

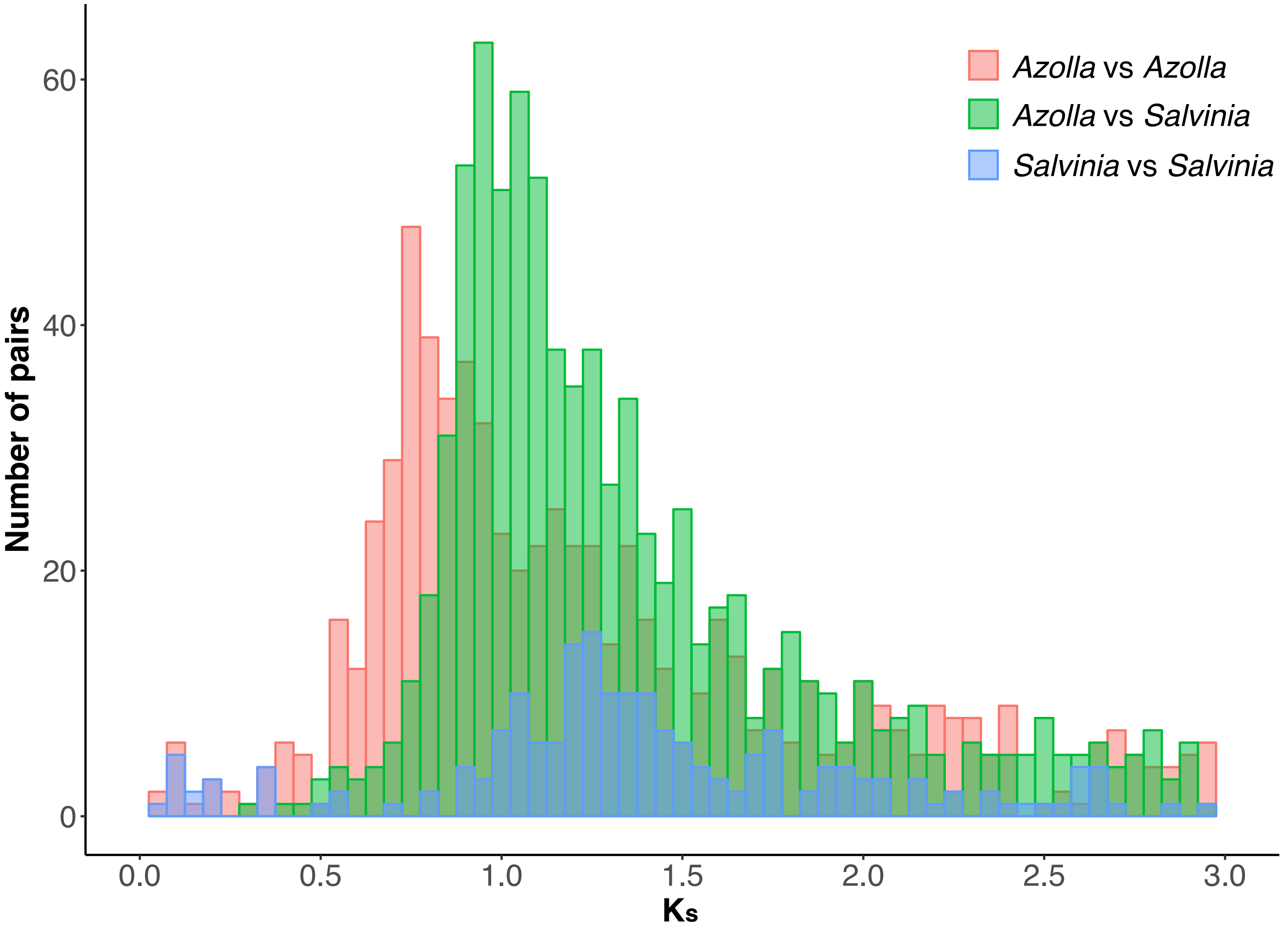

Supplementary Figure 7. Histogram plots of frequency distributions of Ks values estimated from pairs of syntenic paralogs within *Azolla* (red) and *Salvinia* (blue) genomes, as well as of syntenic orthologs between *Azolla* and *Salvinia* (green).

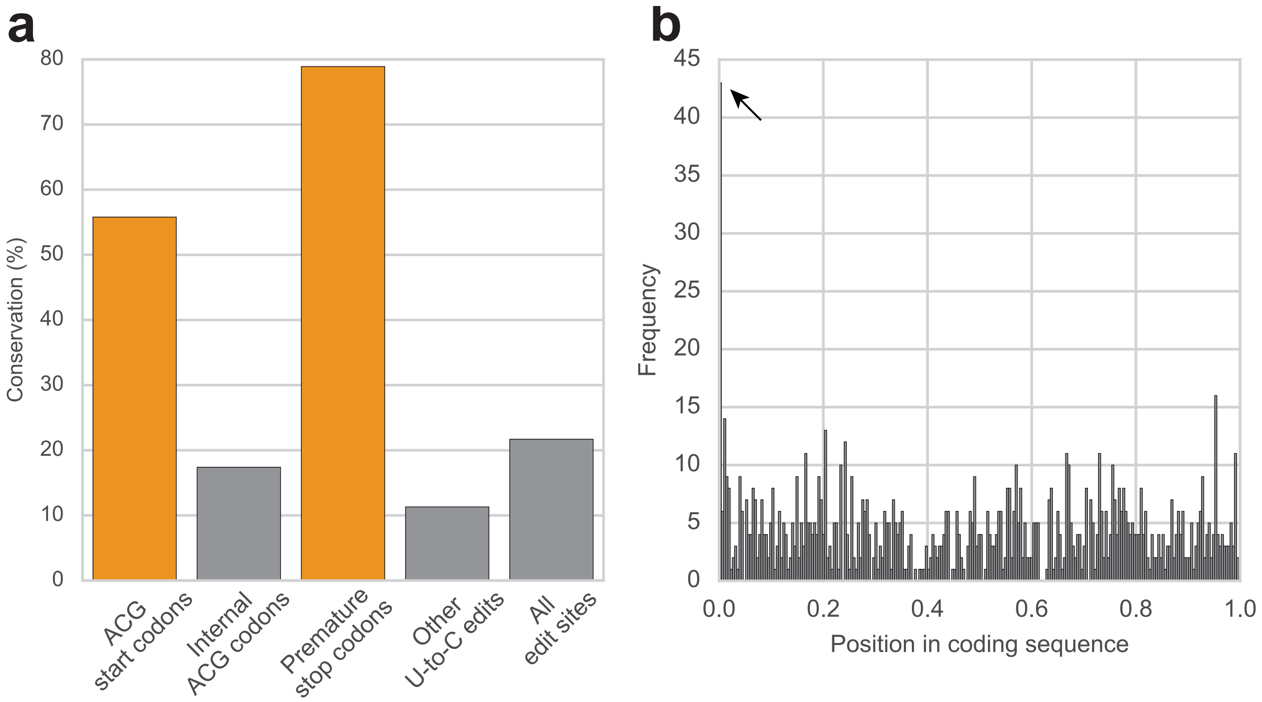

Supplementary Figure 8. Patterns of RNA-editing in *Azolla filiculoides* and *Salvinia cucullata* plastid genomes. (a) High proportions of start and stop codon editing events (orange) are shared between *A. filiculoides* and *S. cucullata*, suggesting that RNA-editing could be a mechanism to control gene expression. (b) RNA-editing sites are concentrated at the start codon (arrow) in plastid protein-coding genes. The x-axis is the relative position in each of the genes, with 0 and 1 being the start and stop codon respectively.

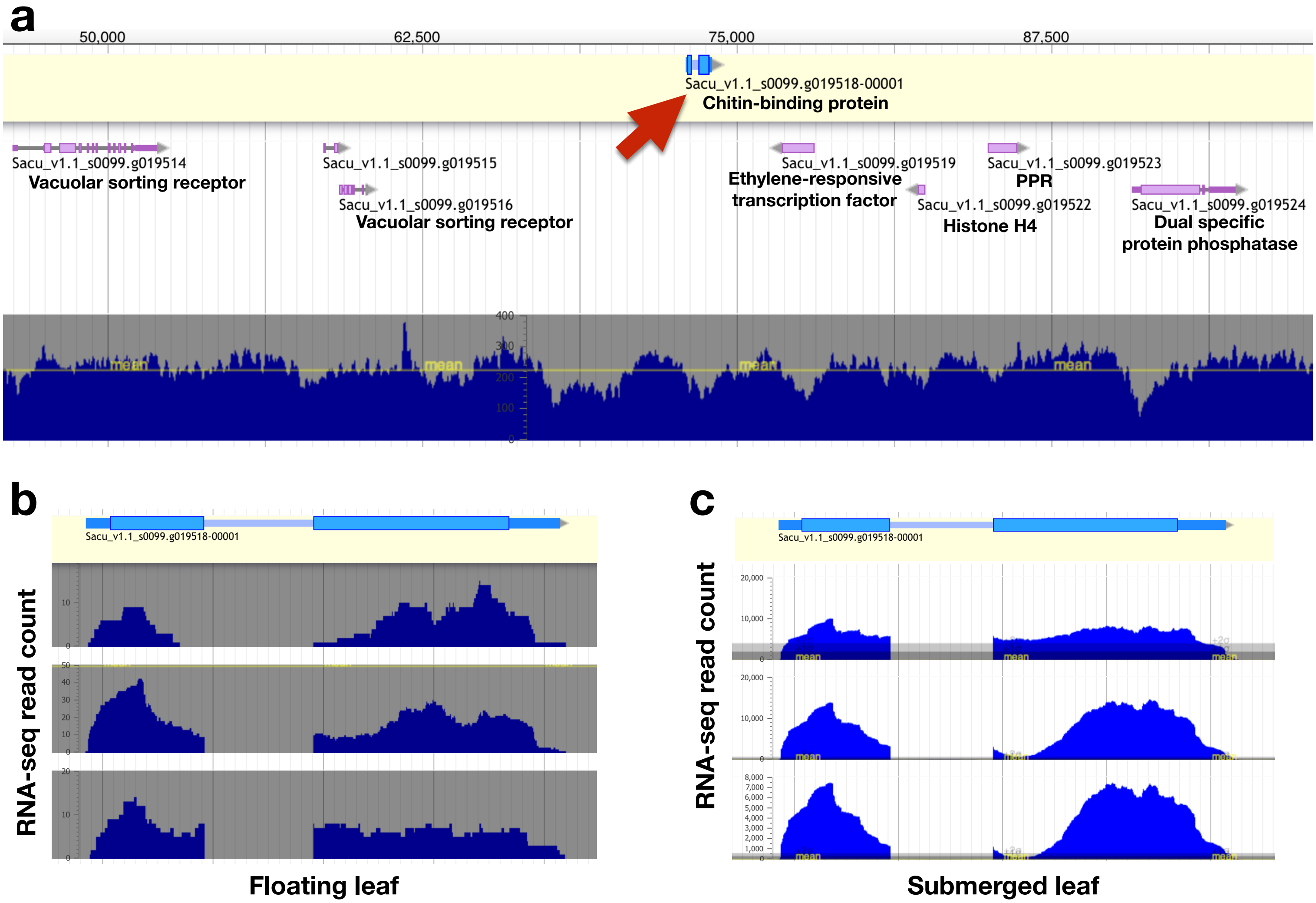

Supplementary Figure 9. *ScTma12* is a nuclear-encoded gene in *Salvinia cucullata* genome. (a) The location of *ScTma12* in scaffold s0099, with up- and down-stream genes all being annotated as plant genes. (b) Expression of *ScTma12* in the floating leaves, and (c) in the submerged leaves. The intron in *ScTma12* is supported by RNA-seq data.

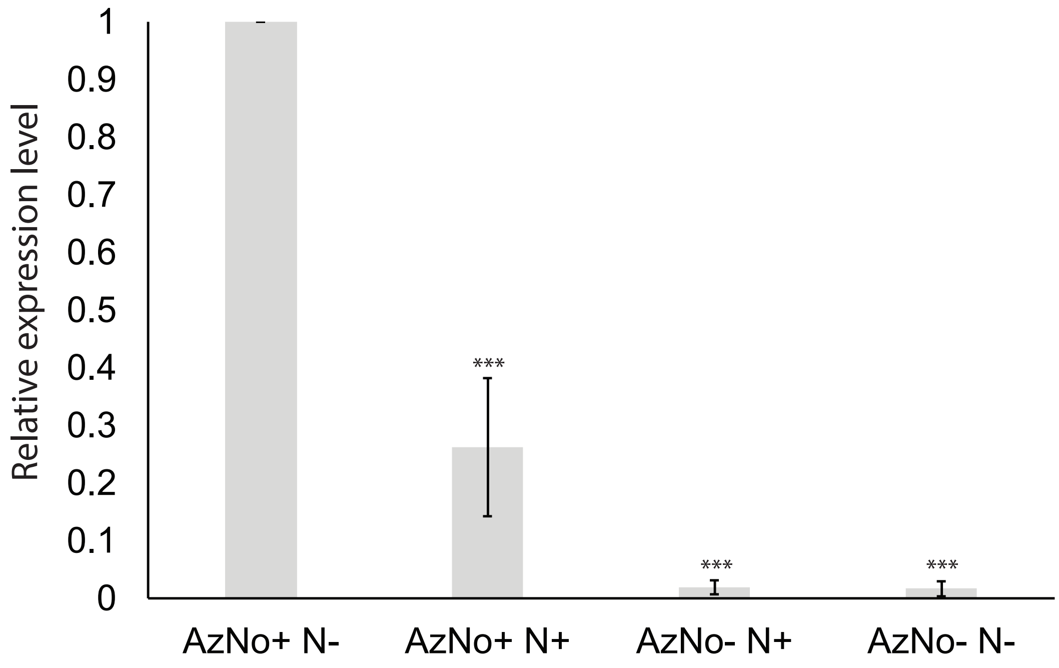

Supplementary Figure 10. Cyanobacterial *NifH* expressions in *Azolla filiculoides* using real-time PCR. Low expression in AzNo- N+/- conditions indicates the cyanobiont was removed, and in AzNo+ N+, that exogenous nitrogen impacts *Nostoc azollae* nitrogenase activity. Asterisk indicates p-value < 0.0001.

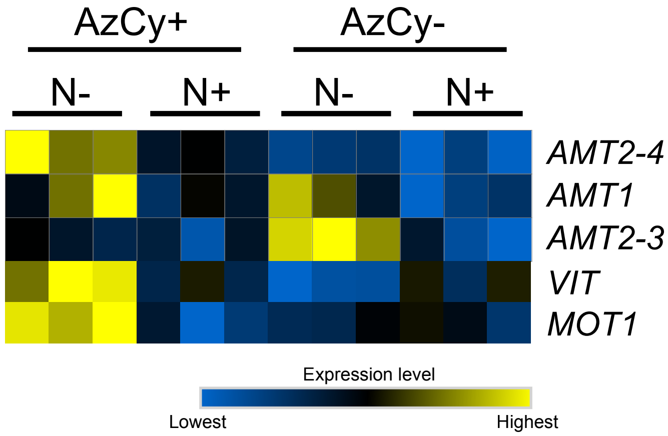

Supplementary Figure 11. Gene expression pattern of selected transporter genes.

Supplementary Figure 12. Phylogeny of squalene-hopene cyclase (SHC). Among streptophytes, SHC homologs can only be found in ferns, lycophytes, mosses, and liverworts, and appear to be absent in seed plants and in green algae. Plant SHCs are not monophyletic, and are interspersed among bacterial sequences, suggesting multiple horizontal gene transfers might have taken place. Cyanobacteria and heterotrophic bacteria are colored in green and orange, respectively. The numbers above branches are bootstrap (BS) support values (BS=100 is omitted), and the thickened branches indicate BS > 70. Blue asterisks denote the sequences coming from plant whole genome assemblies.

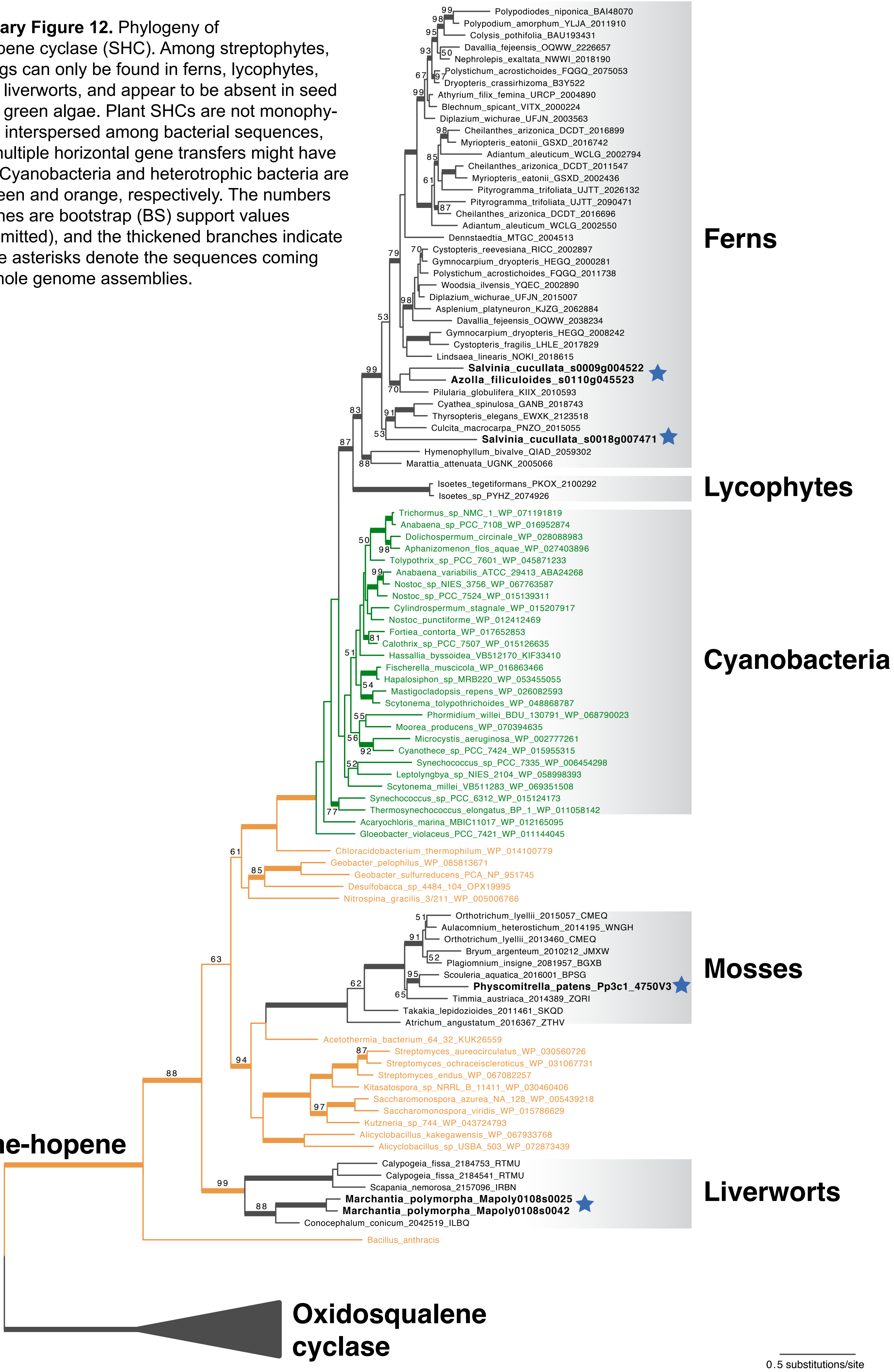

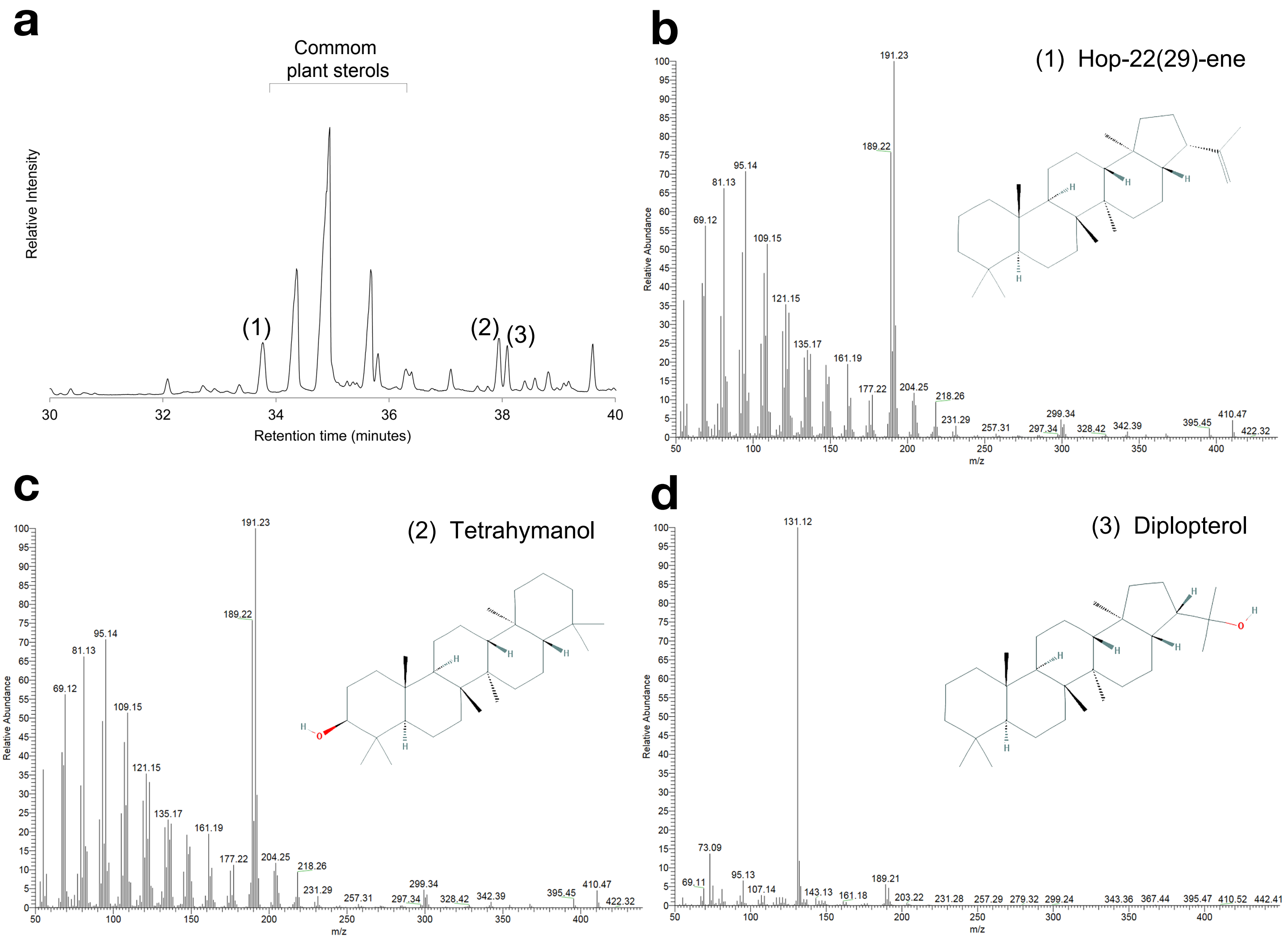

Supplementary Figure 13. Identification of SHC-synthesized triterpenes in *Salvinia cucullata*. (a) Partial GC/MS chromatogram of a total lipid extract of *S. cucullata*, indicating major peaks of common plant sterols (Campesterol, Stigmasterol and β-sitosterol) and peaks 1,2 and 3 representing SHC-synthesized triterpenes. Mass spectra of the identified compounds (b) Hop-22(29)-ene, (c) Tetrahymanol, and (d) 22-hydroxyhopane (diplopterol).

Supplementary Table 1. Species included in this study for flow cytometry and/or genome sequencing.

**Taxon**	**Source**	**Voucher/Accession**
Azolla filiculoides	The Netherlands, Utrecht, Galgenwaard ditch	Dijkhuizen et al 2018
Azolla rubra	International Rice Research Institute	IRRI 6502
Azolla microphylla	International Rice Research Institute	IRRI 4021
Azolla mexicana	International Rice Research Institute	IRRI 2001
Azolla caroliniana 1	International Rice Research Institute	IRRI 3017
Azolla caroliniana 2	International Rice Research Institute	IRRI 3004
Azolla nilotica	International Rice Research Institute	IRRI 5001
Salvinia cucullata	Dr. Cecilia Koo Botanic Conservation Center	K060108
Pilularia americana	Duke University Greenhouse	F.-W. Li s.n. (DUKE)
Regnellidium diphyllum	Taipei Botanic Garden	Wade 4794 (TAIF)
Marsilea crenata	Taipei Botanic Garden	Kuo 4170 (TAIF)

Supplementary Table 2. Genome assembly statistics.

	Genome size (Mb)	Assembled (Mb)	N50 (Kb)	No. scaffold	Average scaffold len (Kb)	% Genomic reads mapped*	% RNA reads mapped
Azolla filiculoides	753	622.6	964.7	3839	162.2	97.14	93.77
Salvinia cucullata	255	231.8	719.8	3721	62.3	95.76	95.85

*contaminated Illumina reads removed before mapping

Supplementary Table 3. Gene annotation statistics. Gene composition in *Azolla* and *Salvinia* by feature type. Abbreviations: Low Confidence (LC), High Confidence (HC).

		Azolla	***Salvinia***
All genes	Count	51098	28968
	Sum length (Mb)	235.2	75.2
	Proportion of assembly	37.8%	32.3%
	Mean transcript length (bp)	821	1282
	Mean number of introns	4.1	4.9
	Mean intron length (bp)	1151	257
LC genes	Count	30897	9054
	Sum length (Mb)	134.2	6.9
	Proportion of assembly	21.6%	3.0%
	Mean transcript length (bp)	476	463
	Mean number of introns	2.6	2.0
	Mean intron length (bp)	2352	284
HC genes	Count	20203	19780
	Sum length (Mb)	101	68.3
	Proportion of assembly	16.2%	29.3%
	Mean transcript length (bp)	1347	1282
	Mean number of introns	5.3	5.2
	Mean intron length (bp)	587	254
tRNA genes	Count	6992	10507
	Sum length (Mb)	0.6	0.9
	Proportion of assembly	0.1%	0.4%
rRNA genes	Count	1397	1161
	Sum length (Mb)	1.6	1.7
	Proportion of assembly	0.3%	0.7%

Supplementary Table 4. Repeat annotation results. Genome composition by number or elements, sum length, and proportion of assembly.

		Azolla	***Salvinia***
Repeats	Sum length (Mb)	333.6	103.7
	Proportion of assembly	53.6%	44.5%
RNA Transposons	Sum length (Mb)	239.1	47.8
	Proportion of assembly	47.0%	26.2%
DNA Transposons	Sum length (Mb)	15.0	5.4
	Proportion of assembly	2.4%	2.3%
Satellite	Sum length (Mb)	16.1	13.6
	Proportion of assembly	2.6%	5.8%
