# Supplementary material for: Fern genomes elucidate land plant evolution and cyanobacterial symbioses
Source: Nat Plants. 2018 Jul 2;4(7):460–72. doi: 10.1038/s41477-018-0188-8 (PMC6786969; doi:10.1038/s41477-018-0188-8)
Supplement: Supplementary file 2 — Reporting Summary [file 41477_2018_188_MOESM2_ESM.pdf]

nature research

Corresponding author(s): Fay-Wei Li

Reporting Summary

Nature Research wishes to improve the reproducibility of the work that we publish. This form provides structure for consistency and transparency in reporting. For further information on Nature Research policies, see Authors & Referees and the Editorial Policy Checklist.

Statistical parameters

When statistical analyses are reported, confirm that the following items are present in the relevant location (e.g. figure legend, table legend, main text, or Methods section).

n/a	Confirmed	
☐	☒	The exact sample size (*n*) for each experimental group/condition, given as a discrete number and unit of measurement
☐	☒	An indication of whether measurements were taken from distinct samples or whether the same sample was measured repeatedly
☐	☒	The statistical test(s) used AND whether they are one- or two-sided<br>*Only common tests should be described solely by name; describe more complex techniques in the Methods section.*
☒	☐	A description of all covariates tested
☐	☒	A description of any assumptions or corrections, such as tests of normality and adjustment for multiple comparisons
☐	☒	A full description of the statistics including central tendency (e.g. means) or other basic estimates (e.g. regression coefficient) AND variation (e.g. standard deviation) or associated estimates of uncertainty (e.g. confidence intervals)
☐	☒	For null hypothesis testing, the test statistic (e.g. *F*, *t*, *r*) with confidence intervals, effect sizes, degrees of freedom and *P* value noted<br>*Give P values as exact values whenever suitable.*
☒	☐	For Bayesian analysis, information on the choice of priors and Markov chain Monte Carlo settings
☒	☐	For hierarchical and complex designs, identification of the appropriate level for tests and full reporting of outcomes
☒	☐	Estimates of effect sizes (e.g. Cohen's *d*, Pearson's *r*), indicating how they were calculated
☐	☒	Clearly defined error bars<br>*State explicitly what error bars represent (e.g. SD, SE, CI)*

Our web collection on statistics for biologists may be useful.

Software and code

Policy information about availability of computer code

Data collection	*Provide a description of all commercial, open source and custom code used to collect the data in this study, specifying the version used OR state that no software was used.*
Data analysis	We employed a number of software for data analyses in this study, which was described in detail in the materials and methods, including the parameters used, versions (if applicable), and citations.

For manuscripts utilizing custom algorithms or software that are central to the research but not yet described in published literature, software must be made available to editors/reviewers upon request. We strongly encourage code deposition in a community repository (e.g. GitHub). See the Nature Research guidelines for submitting code & software for further information.

Data

Policy information about availability of data

All manuscripts must include a data availability statement. This statement should provide the following information, where applicable:

- Accession codes, unique identifiers, or web links for publicly available datasets
- A list of figures that have associated raw data
- A description of any restrictions on data availability

The genome assemblies and annotations can be found in www.fernbase.org. The raw genomic and transcriptomic reads generated in this study were deposited in NCBI SRA under the BioProject PRJNA430527 and PRJNA430459. The sequence alignments and tree files can be found in Supplementary Data.

Field-specific reporting

Please select the best fit for your research. If you are not sure, read the appropriate sections before making your selection.

☒ Life sciences ☐ Behavioural & social sciences ☐ Ecological, evolutionary & environmental sciences

For a reference copy of the document with all sections, see nature.com/authors/policies/ReportingSummary-flat.pdf

Life sciences study design

All studies must disclose on these points even when the disclosure is negative.

Sample size	For phylogenetic analyses, bootstrapping datasets were sampled between 100 to 1000 times, which is the field standard.
Data exclusions	Gene models without transcript or homology supports were excluded (see the supplementary discussion).
Replication	The RNA-seq experiments were done with three biological replicates per treatment.
Randomization	The plant cultures for RNA-seq were placed on the same growth chamber shelf, but the positions were randomized in terms of nutrient treatments and symbiont types.
Blinding	Blinding is not applicable in this study.

Reporting for specific materials, systems and methods

Materials & experimental systems

n/a	Involved in the study	
☐	☒	Unique biological materials
☒	☐	Antibodies
☒	☐	Eukaryotic cell lines
☒	☐	Palaeontology
☒	☐	Animals and other organisms
☒	☐	Human research participants

Methods

n/a	Involved in the study	
☒	☐	ChIP-seq
☐	☒	Flow cytometry
☒	☐	MRI-based neuroimaging

Unique biological materials

Policy information about availability of materials

Obtaining unique materials	The plant materials used in this study are available upon request (to F.-W. Li or H. Schluepmann)

Flow Cytometry

Plots

Confirm that:

☐ The axis labels state the marker and fluorochrome used (e.g. CD4-FITC).

☐ The axis scales are clearly visible. Include numbers along axes only for bottom left plot of group (a 'group' is an analysis of identical markers).

☐ All plots are contour plots with outliers or pseudocolor plots.

☒ A numerical value for number of cells or percentage (with statistics) is provided.

Methodology

Sample preparation

We used flow cytometry to estimate the genome sizes of Pilularia americana, Regnellidium diphyllum, Marsilea minuta, Salvinia cucullata.

1. Prepare buffer for use.
  a. Allocate appropriate amount of Backmen stock buffer to a 50-ml tube based on an estimation of 1-1.5 ml per sample.
  b. Add 0.04 g PVP-40, 5 µl 2-mercaptoethanol, 1 µl RNase per ml of buffer.
2. Extract sample and standard nuclei by chopping leaf tissue
  a. Add 500 µl of buffer to a glass Petri dish.
  b. Add a (~400 mm2) piece of young leaf to the Petri dish, and chop it with a razor on ice until most tissue slices are less than 1 mm in size.
  c. Filter the chopped sample and standard into a 2.0-ml tube through a 30-µm nylon mesh.
  d. Add additional buffer to the sample, and ensure that the filtered leaf nuclei solution is greater than 500 µl in volume or more depending on need.
3. Staining nuclei solutions
  a. Mix sample nuclei and standard leaf nuclei solutions into a 500-µl volume in 2.0-ml tubes.
  b. Add 10 µl PI solution (2.04 mg/ml) into each of mixed nuclei solutions.
  c. Incubate in the dark at 4 °C for 1 h for staining.

Recipes
Backmen stock buffer
1.0% Triton X-100
50 mM Na2SO3
50 mM Tris-HCl (pH 7.5)
ddH2O (the solvent)
Note: Store at 4 °C up to 1 year.

Instrument

BD FACSCan system (BD Biosciences, Franklin Lake, NJ, USA)

Software

BD FACSCan system (BD Biosciences, Franklin Lake, NJ, USA)

Cell population abundance

Pilularia americana:
Replicate 1: sample peak particle number = 1514, standard1 peak particle number = 1154.
Replicate 2: sample peak particle number = 1834, standard1 peak particle number = 1371.
Replicate 3: sample peak particle number = 1450, standard1 peak particle number = 1036.

Regnellidium diphyllum:
Replicate 1: sample peak particle number = 1222, standard1 peak particle number = 1737.
Replicate 2: sample peak particle number = 1180, standard1 peak particle number = 1613.
Replicate 3: sample peak particle number = 1137, standard1 peak particle number = 1759.

Marsilea minuta:
Replicate 1: sample peak particle number = 1892, standard1 peak particle number = 1118.
Replicate 2: sample peak particle number = 1850, standard1 peak particle number = 1209.
Replicate 3: sample peak particle number = 1892, standard1 peak particle number = 1227.

Salvinia cucullata:
Replicate 1: sample peak particle number = 1084, standard1 peak particle number = 1484, standard2 peak particle number = 1170.
Replicate 2: sample peak particle number = 1129, standard1 peak particle number = 1552, standard2 peak particle number = 1253.
Replicate 3: sample peak particle number = 1229, standard1 peak particle number = 1584, standard2 peak particle number = 1500.

Gating strategy

For particle acquisition, we set a threshold of FL2-H = 52 for the samples of Pilularia americana, Regnellidium diphyllum, and Marsilea minuta. For Salvinia cucullata, a threshold of FL2-H = 100 is applied.

☐ Tick this box to confirm that a figure exemplifying the gating strategy is provided in the Supplementary Information.
